# Cyclic mechanical stretching enhances mitophagy and oxidative stress resistance in adipose-derived stem cells via the Piezo1/ATP axis to accelerate wound healing

**DOI:** 10.7150/thno.118364

**Published:** 2025-09-08

**Authors:** Yujie Xiao, Zhijun Shi, Yixuan Yuan, Danna Yao, Rongqin Feng, Yue Zhang, Deli Zhao, Hao Zhang, Panpan Sun, Yang Liu, Yan Li, Xuefeng Shen, Zhantong Wang, Dahai Hu, Hao Guan, Hongtao Wang

**Affiliations:** 1 Department of Burns and Cutaneous Surgery, Xijing Hospital, Fourth Military Medical University, 127 Changle West Road, Xi'an 710032, China.; 2 Laboratory for Multiscale Mechanics and Medical Science, Department of Engineering Mechanics, State Key Laboratory for Strength and Vibration of Mechanical Structures, School of Aerospace Engineering, Xi'an Jiaotong University, Xi'an 710049, China.; 3 Xijing 986 Hospital Department, Fourth Military Medical University, Xi'an, Shaanxi, China.; 4 Northwest University, Xi'an 710069, China.; 5 Department of Occupational and Environmental Health and the Ministry of Education Key Lab of Hazard Assessment and Control in Special Operational Environment, School of Public Health, Fourth Military Medical University, China.

**Keywords:** adipose-derived stem cells, cyclic mechanical stretch, Piezo1, mitophagy, oxidative stress

## Abstract

Adipose-derived stem cells (ADSCs) hold significant potential in regenerative medicine, yet their therapeutic efficacy is often limited by low survival rates in the presence of oxidative stress. While mechanical cues regulate cytoskeletal dynamics, their roles in modulating cellular metabolism and mitochondrial adaptation remain unexplored. This study aimed to elucidate how physiological-range cyclic mechanical stretching (CMS) enhances ADSCs resistance to oxidative stress through the Piezo1/ATP signaling axis, thereby establishing an innovative strategy for developing antioxidant-functionalized stem cell therapies.

**Methods:** To examine the impact of CMS on oxidative stress resistance, ADSCs were exposed to CMS (8% strain, 0.5 Hz, 24 h) using the Flexcell FX-6000 system. Oxidative stress models employed H₂O₂ (200 μM), with apoptosis, mitochondrial function, and metabolic flux analyzed *in vitro*. A murine full-thickness wound model was used to assess *in vivo* survival and regenerative outcomes.

**Results:** CMS activated Piezo1 channels, resulting in enhanced ATP synthesis and remodeling of the tricarboxylic acid cycle. This improved the effectiveness of mitochondrial oxidative phosphorylation. Mechanically preconditioned ADSCs exhibited reduced apoptosis, ​enhanced oxidation resistance, stabilized mitochondrial membrane potential, and upregulated mitophagy. *In vivo*, these cells demonstrated superior healing capacity and accelerated wound closure.

**Conclusion:** CMS orchestrated the Piezo1/ATP-driven metabolic-mitochondrial axis to enhance ADSCs oxidative stress resistance by coupling metabolic reprogramming with mitophagy activation. This mechanometabolic interaction identifies mechanical signaling as a direct regulator of cellular bioenergetics, offering a translatable strategy to engineer antioxidant-functionalized stem cells for regenerative therapies.

## Introduction

Wound healing presents multifaceted challenges, particularly in complex wounds such as diabetes ulcer and post-traumatic infections, where delayed tissue regeneration remains prevalent 1,2. Current therapeutic strategies predominantly target anti-infection, inflammation modulation, repair cells proliferation, and collagen realignment 3. Growth factors such as transforming growth factor-beta (TGF-β) and vascular endothelial growth factor (VEGF) stimulate cellular proliferation and angiogenesis 4, whereas gene therapy targets to suppress pro-inflammatory cytokines such as interleukin-1 (IL-1) and tumor necrosis factor-alpha (TNF-α) 5. Mesenchymal stem cells (MSCs) exhibits potential in vascularization and immunomodulation via differentiation and paracrine signaling among regenerative interventions 6.

Adipose-derived stem cells (ADSCs) are particularly promising for skin repair due to their accessibility, low immunogenicity, and multipotency 7. Their therapeutic potential relies on two key mechanisms: paracrine secretion of pro-angiogenic (VEGF and fibroblast growth-2 [FGF2]) and immunomodulatory factors (IL-10) to promote vascularization, collagen remodeling, and mitochondrial transfer to directly rejuvenate damaged cells 8. However, under pathological conditions, such as burns and diabetic ulcers, elevated levels of reactive oxygen species (ROS) induce oxidative stress that compromises ADSCs viability and function. ROS not only compromises mitochondrial integrity and induces DNA fragmentation in stem cells, but also damages the extracellular matrix (ECM), undermining ADSCs differentiation and paracrine capacity 9. Although advanced biomaterials mimic the physicochemical properties of the native ECM, they remain inadequate for counteracting the persistent oxidative damage inflicted by ROS on stem cells 10. This limitation highlights an immediate necessity for advanced therapeutic strategies that integrate ROS-scavenging functionalities with ECM-mimetic systems to preserve stem cells viability and enhance regenerative efficacy under pathological oxidative stress. This underscores a critical challenge in regenerative medicine, imparting metabolic resilience to transplanted cells against oxidative stress while preserving their paracrine and regenerative functions. Conventional antioxidant strategies (e.g., CSF2RB overexpression) transiently alleviate oxidative damage and disrupt endogenous signaling 11, highlighting the urgent need for non-genetic approaches to systemically enhance ADSCs oxidative stress resistance.

Preconditioning strategies, including hypoxia and mechanical stimulation, have been shown to enhance specific functions and improve therapeutic efficacy 12,13. Mechanical signals are key regulators of stem cell-microenvironment interactions, influencing phenotypic remodeling, inflammation modulation, and tissue regeneration 14. In ADSCs, mechanical cues can enhance paracrine secretion of VEGF and basic FGF, thereby promoting angiogenesis and collagen secretion to facilitate skin regeneration and expansion 15,16. Furthermore, emerging evidence suggests that mechanical stretched ADSCs-conditioned medium induces anti-inflammatory M2-like macrophage polarization, thereby accelerating chronic wound healing and tissue repair 17. Collectively, these findings highlight the sensitivity of ADSCs to mechanical stimuli and their ability to translate biomechanical cues into regenerative outcomes.

The core of this mechanotransduction process is the Piezo1 channel—an evolutionarily conserved cation channel that directly transduces physical signals of membrane tension into biochemical signals 18. Emerging evidence positions Piezo1 as a master regulator of stem cells fate: In MSCs, mechanical activation of Piezo1 regulates osteogenic differentiation and sustains stemness through Ca²⁺-dependent pathways 19-21.

Crucially, in addition to the plasma membrane, Piezo1 may also be localized to the mitochondrial associated membrane (MAM), where it facilitates IP3R-mediated Ca²⁺ transfer to mitochondria, regulating tricarboxylic acid (TCA) circulating flux and ATP synthesis 22,23. Notably, mechanical stretching-activated Piezo1 signaling has been shown to remodel cellular metabolism, including enhanced TCA circulating flux and energy (ATP) production 24,25. This dual role in mechanistic induction and metabolic reprogramming makes Piezo1 a key mediator of biomechanical-metabolic coupling.

Beyond In addition to its role in energy metabolism, ATP functions also act as a purinergic signaling molecule via P2X7 receptors activating the AMPK/mTOR pathway, and balancing autophagy and anabolism 26,27. Thus, the Piezo1/ATP axis provides a novel framework for dissecting how physical forces dictate stem cells fate. However, its spatiotemporal dynamics in ADSCs oxidative stress resistance remain unexplored.

In oxidative environments, pathological reactive oxygen species (ROS) open mitochondrial permeability transition pores, triggering cytochrome *c* release and apoptosis. Within this context, mitophagy—the selective removal of damaged mitochondria via PINK1/Parkin-dependent pathways—is essential for maintaining redox homeostasis 28,31,32. It counteracts damage by ubiquitinating depolarized mitochondria for autophagic degradation. Intriguingly, mitophagy activation extends beyond hypoxia-inducible factor 1-alpha (HIF-1α) signaling, with mechanical cues and key metabolites like ATP emerging as critical regulators 30,33. However, whether mechanical forces engage mitophagy to enhance the metabolic fitness of ADSCs remains unclear.

In this study, we investigated whether CMS activates the Piezo1/ATP signaling axis to regulate mitophagy and antioxidant defenses, enhancing ADSCs survival and function in oxidative microenvironments. Using *in vitro* assays and murine wound models, we evaluated whether mechanically preconditioned ADSCs exhibit improved survival and reparative capacity driven by ATP-fueled mitochondrial optimization and amplified paracrine secretion. Our study provides insights into a mechanobiological paradigm in which physical stimuli "train" stem cells to resist metabolic stress, offering a blueprint for next-generation regenerative therapies.

## Methods

### Ethics statement

The Institutional Animal Care and Use Committee (IACUC) of the Air Force Medical University (AFMU) provided ethical approval for the animal experiments (permit no. 20231002). All procedures strictly followed the institution's animal welfare protocols 34, with rigorous measures implemented to reduce animal distress. Surgical interventions were performed under isoflurane-induced anesthesia to ensure humane treatment throughout the study.

### Cell culture

Adipose tissue was collected from patients undergoing abdominal liposuction from the Department of Burn and Cutaneous Surgery at Xijing Hospital. All patients provided written informed consent to participate and to allow use of their tissues for research purposes. This study complies with the Declaration of Helsinki and was approved by the Ethics Committee of Xijing Hospital (KY20242078-F-1). ADSCs were harvested using the established isolation protocols 35,36. Briefly, adipose tissue specimens were rinsed in sterile phosphate-buffered saline (PBS, 1% penicillin-streptomycin) and mechanically minced into 1-mm³ fragments. Tissue digestion was performed using 1 mg/mL collagenase type I (Sigma-Aldrich, C1-BIOC, USA) under continuous agitation at 37 °C for 60 min. The resulting cell suspension was sequentially filtered through a 100 μm nylon mesh and subjected to centrifugation (300×g, 5 min). Pelleted cells were resuspended in ADSCs-specific growth medium (Cyagen Biosciences, HUXMD-90011, China) supplemented with 10% fetal bovine serum, 100 U/mL penicillin, 100 μg/mL streptomycin, and then seeded into 25 cm² culture flasks. Cells were maintained at 37 °C in a 5% CO₂ humidified incubator, with complete medium replacement performed 24 h post-seeding. Morphological assessment was conducted using inverted phase-contrast microscopy, and subculturing was routinely executed at an interval of 3-4 days upon reaching 80-90% confluence. For phenotypic validation, third-passage ADSCs were analyzed via flow cytometry (BD FACS Calibur™, Becton-Dickinson, USA) to quantify the expression of surface markers including CD31, CD105, CD11b, CD34, CD44, and CD90.

### *In vitro* differentiation of ADSCs into adipocytes and osteoblasts

*In vitro* differentiation of ADSCs into adipocytes and osteoblasts. Passage 4 ADSCs were seeded at a density of 5×10³ cells/cm² into six-well plates precoated with cover slips. To induce adipogenic differentiation, cells were cultured using adipogenic differentiation medium (Cyagen Biosciences Inc, Guangzhou, China), and for osteogenic differentiation, osteogenic differentiation medium (Cyagen Biosciences Inc.) was used. The differentiation process was carried out for three weeks. After differentiation, cells were fixed with 10% formalin and then stained with Oil Red O for adipogenic differentiation or Alizarin Red for osteogenic differentiation, following the manufacturer's protocols (Cyagen Biosciences Inc.). After staining, cells were washed three times with PBS, and the results were observed and recorded using a microscope (Olympus).

### Application of mechanical stretch preconditioning

**Figure 1:**
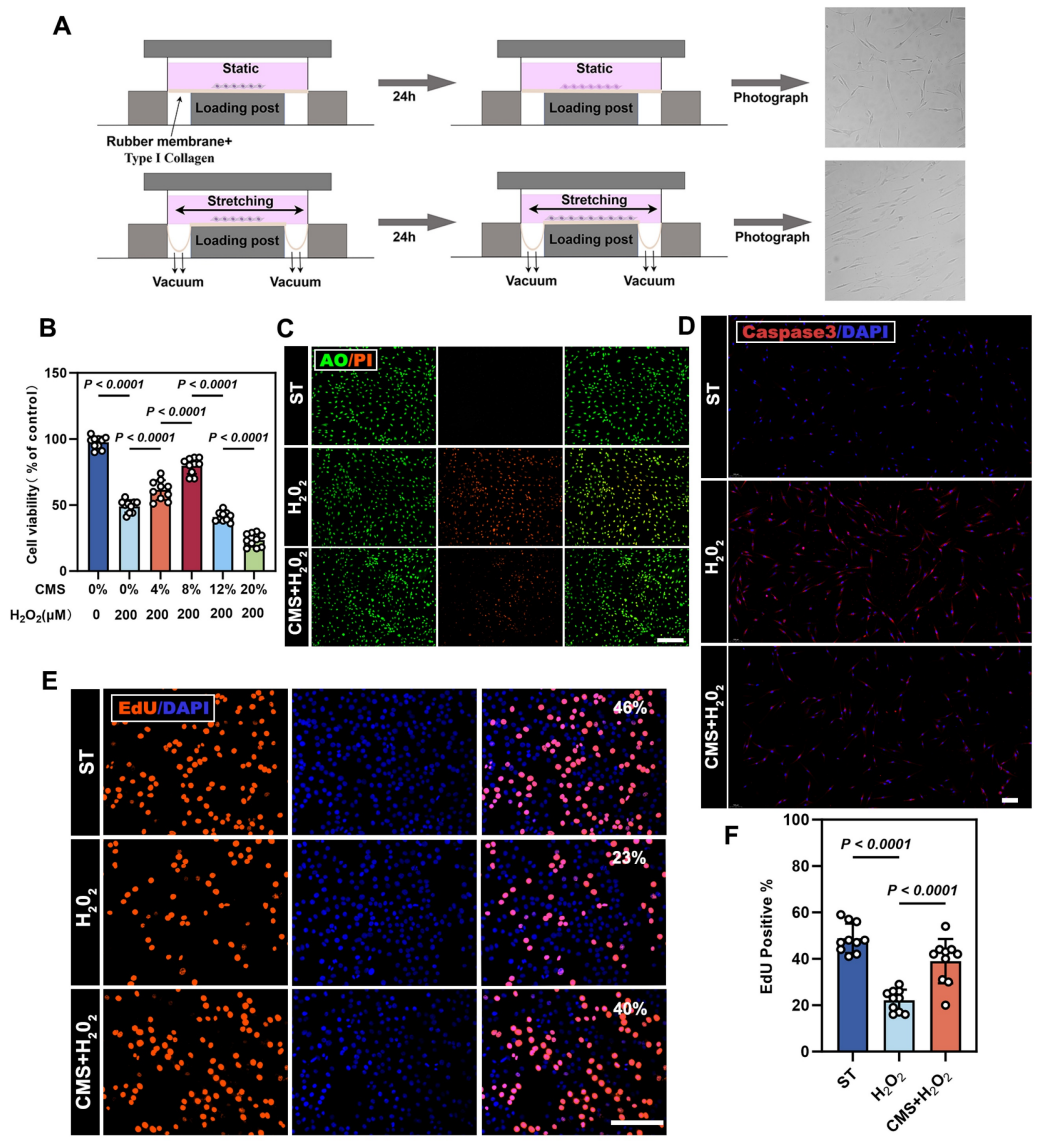
** Cyclic mechanical stretch (CMS) enhances adipose-derived stem cells (ADSCs) survival under oxidative stress.** (A) Schematic diagram of the Flexcell Tension System for cyclic mechanical stimulation. (B) Protective effect of different strain-preconditioned ADSCs against H_2_O_2_-induced cytotoxicity (n = 10). (C) AO/PI, live/dead staining (green: viable; red: apoptotic cells), (Scale bar= 275 μm). (D) Immunofluorescence (IF) of caspase-3 (red) (Scale bar = 100 μm,). (E) 5-ethynyl-2′-deoxyuridine (EdU) assay (red: proliferating cells; blue: DAPI) comparing proliferation among the ST, H_2_O_2_-treated, and preconditioned + H_2_O_2_ groups (Scale bar = 125 μm). (F) Quantification of EdU + cells (%) using GraphPad Prism 10.1(n = 10). (Data are presented as mean ± standard deviation).

Mechanical stretching of ADSCs was performed using the FX-6000T™ Flexcell Tension Plus system (Flexcell International Corporation, Hillsborough, NC, USA). ADSCs were seeded onto six-well BioFlex™ culture plates precoated with collagen I, featuring flexible silicone membranes for controlled strain applications. After cell adhesion was established, a cyclic mechanical strain was applied to the experimental group using a sinusoidal waveform pattern. Control group cells were cultured on identical BioFlex™ plates but maintained under static (non-stretched) conditions. This system enabled the application of uniform mechanical perturbations through tunable substrate stiffness (0.1-80 kPa range) and covalently bonded extracellular matrix coatings (Figure 1A).

### Cell viability assay

To assess the cytotoxicity of the mechanically stimulated ADSCs, cell viability was quantified using a Cell Counting Kit-8 (CCK-8; Beyotime, China). ADSCs were seeded onto 96-well plates at a density of 1 × 10⁴ cells/well and maintained under standard culture conditions (37 °C, 5% CO₂). According to the manufacturer's protocol, 10 µL of CCK-8 reagent was added to each well, and the plates were incubated for 1 h at 37 °C. Absorbance was measured at 450 nm using an Infinite M200 Pro microplate reader (Tecan, Switzerland). ADSCs cultured under normoxic, non-stretched conditions served as the baseline control.

### Mito-tracker red staining

To visualize mitochondrial and nuclear structures, ADSCs underwent co-staining with Mito-Tracker Deep Red FM (Beyotime, China) and Hoechst 33342 (Beyotime, China) through a 30-min incubation at 37 °C. Following this dual-labeling procedure, cellular specimens were subjected to PBS rinses before transitioning to an ADSCs-optimized culture medium.

### Immunofluorescence analysis

Third-passage ADSCs from the experimental group were fixed in 4% paraformaldehyde (PFA) for 20 min, followed by two rounds of Triton X-100-mediated permeabilization cycles (0.5% in PBS, 2×20 min) and 10% BSA (1 h, room temperature) blockade. Specimens were subsequently immunolabeled with primary antibodies (AccuRef Scientific, China, 1:100): Bax (Proteintech, China), Bcl-2 (Proteintech), Caspase-3 (Proteintech), GPX4 (Abcam), 4HNE (Abcam), and Piezo1 (Proteintech) under 4 °C overnight incubation. After PBS rinses, the samples were treated with fluorescein isothiocyanate (FITC)-conjugated secondary antibody (Bioss,1:400) for 1 h. Nuclei were counterstained with DAPI (10 min) and imaged using fluorescence microscopy (Olympus).

For mitochondrial-Piezo1 co-localization, live ADSCs pre-stained with Mito-Tracker Red (Beyotime, China, 30 min) underwent mild fixation (0.5% PFA, 20 min), permeabilized (0.2% Triton-X100, 20 min), and blocked with goat serum (Boster, AR1009). Cells were incubated with Piezo1 primary antibody (Proteintech) followed by Alexa Fluor 488 conjugated secondary antibody (Abcam). Nuclear Labeling was performed using Hoechst 33342 (Beyotime, China).

### Assessment of oxygen consumption rate (OCR)

Mitochondrial respiratory function was quantified using the Seahorse XF96 flux analyzer (Agilent Technologies, Santa Clara, CA, USA) with XF Cell Mito Stress Test Kit. ADSCs were plated in 96-well assay plates at 2×10^4^ cells/well density. Pharmacological modulators were administered in staged injections per metabolic profiling protocol: oligomycin (ATP synthase inhibitor, 1.5 μM) → FCCP (mitochondrial uncoupler, 1 μM) → rotenone/antimycin A (ETC complex I/III inhibitors, 0.5 μM). After the Seahorse analysis, the cells were rinsed with PBS and the cells were counted with a cell counting instrument.

### 5-Ethynyl-2′-Deoxyuridine (EdU) incorporation assay

To assess the effect of mechanical stimulation on ADSCs proliferation, we performed an EdU-based proliferation analysis. ADSCs were seeded onto six-well plates (5×10⁴ cells/well) and cultured at 37 °C. ​Post-adhesion, 500 μL of EdU solution (Beyotime) was added for 2 h incubation. The cells were ​fixed with 4% PFA (20 min)​ and ​permeabilized with 0.5% Triton X-100 (10 min). A ​click reaction mixture​ was applied for 30 min to label proliferating cells, followed by PBS washes. Nuclei were counterstained with Hoechst 33342 in the dark. EdU-positive nuclei were ​visualized using fluorescence microscopy (Olympus)​ and analyzed using ImageJ software (NIH, USA).

### Propidium iodide (PI)/acridine orange (AO) staining

To evaluate the impact of oxidative stress on apoptosis in mechanically prestimulated ADSCs, a PI/AO dual-staining apoptosis assay was performed using the PI/AO Apoptosis Detection Kit (Beyotime). Cells from each group were rinsed with PBS, harvested using EDTA-free trypsin, and resuspended in 100 μL 1X binding buffer. Subsequently, 5 μL of AO and 5 μL of PI were added, and the samples were incubated for 15 min in the dark (Olympus). Apoptotic cells were analyzed through fluorescence microscopy and quantified using ImageJ software (NIH, USA).

### JC-1 assay for mitochondrial membrane potential

To evaluate mitochondrial function in ADSCs following mechanical prestimulation under oxidative stress, we used the JC-1 fluorescent probe (Beyotime). In healthy mitochondria with intact membrane potential (ΔΨm), JC-1 forms red fluorescent aggregates. In contrast, mitochondrial depolarization (a hallmark of dysfunction) causes JC-1 to shift to green, fluorescent monomers. Post-treatment, ADSCs were imaged via fluorescence microscopy (Olympus), and the green/red fluorescence ratio was quantified with ImageJ software (NIH, USA) to assess ΔΨm changes associated with oxidative stress-induced mitochondrial damage.

### ROS, Mito-SOX, and antioxidative activities assays

ROS levels were measured using DCFH-DA oxidation (ROS detection kit, Beyotime), and mitochondrial superoxide was detected using Mito-SOX Red mitochondrial superoxide detection (Beyotime), both processed per standardized protocols. To assess antioxidant activity, the culture medium supernatant was collected by centrifugation at 12,000×g for 5 min. Ferric ion reducing antioxidant power (FRAP; Nanjing Jiancheng Bioengineering Institute, China) and malondialdehyde (MDA; Nanjing Jiancheng Bioengineering Institute, China) levels were measured using commercial kits (Nanjing Jiancheng Bioengineering Institute) according to the manufacturer's instructions.

### Metabolomics

For metabolomic analysis, ADSCs were cultured under Cyclic Mechanical Stretching (N=3 biological replicates) or Static (N=3 biological replicates). Upon medium removal, cells were immediately flash-frozen in liquid nitrogen. Frozen cells were scraped and lysed in ice-cold 80% methanol. After centrifugation (20,000 × g, 10 min, 4°C), the supernatant was collected, dried, and metabolites were rehydrated in 100 µl of 0.03% formic acid. Following vortexing and centrifugation to remove debris, the supernatant was analyzed by liquid chromatography-tandem mass spectrometry (LC-MS/MS) using a Dionex U3000 UHPLC coupled to a Q Exactive Plus mass spectrometer (Thermo Fisher Scientific). Raw data acquired with Unifi 1.8.1 were processed using Progenesis QI V2.3 software. Differential metabolites were identified based on normalized peak areas, defined as those with variable importance in projection (VIP) scores > 1.0 and statistically significant differences (p < 0.05) determined by two-tailed Student's t-test.

### Flow cytometry

Apoptosis was assessed with an Annexin V-Propidium Iodide Staining Kit (BD Pharmingen™, USA), following the manufacturer's instructions. Intracellular ROS levels were measured using DCFH-DA (Beyotime, China) per the recommended protocol. Flow cytometric analyses were conducted on a BD FACSAria™ III system (USA). ROS levels were quantified by determining the mean fluorescent intensity (MFI).

### ELISA

Levels of b-FGF, HGF, TGF-β (following acid activation), VEGF, IGF-1, and IL-6 in cell culture supernatants were detected using ELISA kits (R&D Systems, Inc).

### Cell mitochondria isolation

Mitochondrial fractionation from ADSCs was performed using a commercial isolation kit (Beyotime) following standardized protocols. Post-trypsinization cells underwent room-temperature centrifugation (150×g, 8 min) to obtain pellets, which were then suspended in ice-cold PBS and quantified prior to low-temperature centrifugation (600×g, 5 min, 4 °C). The resulting pellet was treated with protease inhibitor-supplemented isolation buffer (composition: 20 mM HEPES-KOH pH 7.5, 220 mM mannitol, 70 mM sucrose, 1 mM EDTA, 0.5 mM EGTA, and 0.2% w/v fatty acid-free BSA) (PMSF-added) and maintained on ice for 15 min for membrane stabilization. Mechanical disruption was achieved through controlled homogenization (glass homogenizer) until 50% Trypan blue permeability, followed by differential centrifugation: initial clarification (1000×g, 10 min, 4 °C) to remove nuclei/debris, then mitochondrial enrichment via 3500×g centrifugation (10 min, 4 °C). The supernatant representing cytosolic fraction and pelleted mitochondria were separately lysed in PMSF-containing buffer, with protein concentrations quantified via BCA assay (Beyotime). Both mitochondrial and cytosolic lysates were subsequently processed for immunoblotting analysis.

### Western blot assay

Cells were rinsed with ice-cold PBS and processed for protein extraction using two parallel methods: for total protein analysis, cells were lysed in RIPA buffer supplemented with 1% PMSF and 1% protease/phosphatase inhibitor cocktail (Roche, Switzerland) on ice, followed by centrifugation at 12,000×g for 15 min at 4°C to collect supernatants; for mitochondrial enrichment, refer to the mitochondrial separation method described in the previous section. Protein quantification was determined using a BCA assay kit (Heart Biological Technology). Samples were denatured by mixing with 5× loading buffer and boiling for 10 min. Subsequently, 20 μg of proteins per sample was separated on 10% sodium dodecyl sulfate-polyacrylamide gel electrophoresis (SDS-PAGE) gels and transferred onto polyvinylidene fluoride membranes (Roche). Membranes were blocked with 5% skim milk in T-TBS (0.05% Tween-20, 50 mM Tris, 150 mM NaCl, pH 7.5) for 1 h at room temperature and probed overnight at 4°C with primary antibodies against:

For total lysates: Bax (Proteintech), Bcl-2 (Proteintech), GPX4 (Abcam), 4HNE (Abcam), Nrf-2 (Abcam), HO-1 (Abcam), P62 (Proteintech), Piezo1 (Proteintech), β-actin (Proteintech).

For mitochondrial fractions: OPA1 (Biodragon, China), PINK1 (Biodragon, China), Parkin (Biodragon, China), LC3 (Proteintech), TOM20 (Proteintech) (mitochondrial fractions). Following three 10 min washes with T-TBS, the membranes were incubated with horseradish peroxidase-conjugated secondary antibodies (1:5,000, Proteintech) for 1 h at room temperature. Protein bands were visualized using ECL detection reagents (MilliporeSigma) and imaged using a MultiImage Light Cabinet system (Alpha Innotech, San Leandro, CA, United States of America). Quantitative analysis was performed using ImageJ software to determine band intensity.

### Transmission electron microscopy (TEM) analysis

The collected cells were rinsed twice with PBS and fixed in 3% glutaraldehyde at 4 °C for 24 h. Following standard processing protocols involving dehydration, resin embedding, and ultrathin sectioning, as previously described 37, samples were analyzed using a Tecnai G2 Spirit Biotwin transmission electron microscope (FEI; Hillsboro, OR, USA). Thin sections (80 nm) were imaged under optimized conditions with an instrument operating at an accelerating voltage of 100 kV.

### Measurement of ATP, NAD⁺/NADH ratios, and metabolic enzyme activities

Intracellular ATP levels were determined using a bioluminescence assay. Cells were lysed with 100 μL of ATP-releasing reagent (Beyotime) per well, shaken 2 min, and incubated in the dark for 10 min to stabilize luminescence. The lysate was transferred to a black microplate, and ATP content was assessed using a Tecan microplate reader, which detects light emission generated by the luciferin/luciferase enzymatic reaction in an ATP-dependent manner. This method ensures rapid and sensitive quantification of ATP concentrations in cellular samples. Simultaneously, NAD^+^/NADH ratios were analyzed spectrophotometrically using a dedicated detection kit (Beyotime) under standardized reaction conditions. Citrate synthase (CS) and pyruvate dehydrogenase (PDH) activities were both determined colorimetrically using commercial kits (Elabscience, China), according to the manufacturer's instructions. All spectrophotometric data were normalized to total protein concentration (BCA assay).

### Quantitative real-time PCR

Total RNA was isolated using TRIZOL reagent (Invitrogen, USA) through phase separation (chloroform), followed by isopropanol precipitation and 75% ethanol washes. Reverse transcription reactions (20 μL total volume) were assembled with 3 μg RNA, Oligo-dT primers, and components including 5× Reaction Buffer, dNTPs (10 mM), reverse transcriptase, and RNase inhibitor, subjected to thermal cycling: 65 °C/5 min → 42 °C/60 min → 70 °C/5 min. Quantitative PCR amplification was conducted on a StepOnePlus system (Applied Biosystems, Waltham, MA, United States of America) with SYBR Green chemistry (TaKaRa, RR420A), utilizing 20 μL reactions containing 3 μL cDNA, gene-specific primers (0.4 μM each), and SYBR Green Supermix. Thermocycling parameters included initial denaturation (95 °C/5 min), 40 cycles of denaturation (95 °C/5 s) and annealing/extension (60 °C/45 s). All data were normalized to β-actin reference gene expression and analyzed via the 2-ΔΔCT method. Primer sequences are detailed in Supplementary Table 1.

### Small interfering RNA (siRNA) transfection

Piezo1-specific siRNA or scrambled siRNA (control) were sourced from GenePharma, Shanghai, and Lipofectamine 2000 along with Opti-MEM from Thermo Fisher Scientific, USA, were employed for ADSCs. Post-transfection, mRNA expression of Piezo1 was measured at 24 h, whereas protein expression was assessed at 48 h. The siRNA oligonucleotide sequences can be found in Supplementary Table 1.

### Wound healing model

Male Balb/c mice (8 weeks, 20-25 g body weight) were obtained from the Animal Center of Fourth Military Medical University and housed in a controlled environment (22±2 °C, 12/12 h light-dark cycle) with ad libitum access to food and water. To establish a full-thickness cutaneous wound model, the animals were randomly divided into three groups: PBS-treated controls (n=10), ADSCs-treated (n=10), and CMS-ADSCs-treated (n=10). Following anesthesia via isoflurane inhalation, a standardized circular excision (1 cm diameter) was surgically performed on the depilated dorsal surface on day 0. On postoperative day 1, cellular interventions were administered through perilesional multipoint injections (four sites) using either 10⁶ ADSCs/CMS-ADSCs suspended in 100 μL PBS or PBS vehicle alone. Wound healing progression was quantitatively monitored via serial digital imaging (postoperative days 0/3/7/10/14), with wound closure rates calculated using Image Pro Plus 6.0 software, expressed as the percentage of residual wound area relative to baseline area:

Wound closure (%) = [(Day 0 area- Day X area)/Day 0 area] ×100

Terminal tissue sampling on day 11 involved a bloc resection of the healed wounds with adjacent tissues for subsequent histomorphometric analysis.

### Histology

For histological assessment, excised skin tissues were fixed in 4% paraformaldehyde at 4°C for 24 h to preserve structural integrity. Following dehydration through a graded ethanol series and xylene clearing, specimens were embedded in paraffin blocks. Sections (4-5 μm) were prepared using a microtome and stained with hematoxylin and eosin (H&E) for cellular morphology or Masson's trichrome for collagen fiber visualization.

For IF, sections underwent deparaffinization, antigen retrieval in citrate buffer (pH 6.0), and blocking with 5% BSA. Tissues were co-stained overnight at 4°C with primary antibodies against: HLA-A (Abcam), CD31 (Abcam), α-SMA (Abcam). After PBS washes, sections were incubated with Alexa Fluor-conjugated secondary antibodies (Proteintech): goat anti-mouse 488 and goat anti-rabbit 594. Nuclei were counterstained with DAPI. For immunohistochemistry (IHC), sections were processed similarly for antigen retrieval and blocking. Primary antibodies against IL-6 (Abcam) and TNF-α (Abcam) were applied overnight at 4°C. Detection utilized HRP-conjugated secondary antibodies (Abcam) and DAB chromogen (Abcam), followed by hematoxylin counterstaining. All stained sections were imaged using a Pannoramic 250/MIDI slide scanner (3DHISTECH, Budapest, Hungary).

### Statistical analyses

Statistical analyses were conducted using GraphPad Prism 10.0 (GraphPad Software Inc., La Jolla, CA, USA). Experimental data are expressed as mean ± standard error of the mean (SEM) and were obtained from a minimum of three independent experimental replicates. For comparisons involving two experimental groups, the student's *t* test was used to evaluate intergroup differences. When analyzing three or more groups, a one-way analysis of variance was performed, followed by Tukey's post hoc test for multiple comparisons. Statistical significance was defined as a probability value (p-value) below 0.05.

## Results

### CMS enhances oxidative stress resistance and proliferative activity of human ADSCs

CMS with different parameters was applied to ADSCs. Amon them, we found that CMS of an appropriate intensity (8% strain, 24h) significantly promoted the proliferation of ADSCs (Figure S1A, B). Post-stimulation characterization confirmed that CMS-treated ADSCs retained their stem cell identity (Figure S1D), and their differentiation capacity remained normal (Figure S1F). Notably, CMS preconditioning enhanced the secretion of paracrine factors (b-FGF, HGF, TGF-β, VEGF, IGF-1, IL-6), suggesting amplified immunomodulatory and regenerative potential (Figure S1E). This is consistent with previous studies 15,17. An oxidative stress model was established using H₂O₂ concentrations ranging 0-500 μM; 200 μM yielded 50% cell survival rate (IC₅₀) and was subsequently selected for modeling oxidative injury (Figure S1C). To validate the protective efficacy of CMS, the following three groups were designed: (1) Static control (ST) group; (2) H₂O₂-treated group, (200 μM,24 h); and (3) CMS + H₂O₂ group, mechanical preconditioning (8% strain, 0.5 Hz, 24 h) followed by H₂O₂ treatment (200 μM, 24 h). CCK-8 analysis revealed that mechanical preconditioning significantly enhanced the viability of ADSCs under oxidative stress (Figure 1B). Flow cytometry analysis further confirmed that CMS reduced H₂O₂-induced apoptosis (Figure S1G). Consistent with this, PI/AO double staining (Figure 1C) and caspase3 immunostaining (Figure 1D) used for cell death measurement indicated that CMS preconditioning significantly decreased late apoptosis and necrosis. Additionally, EdU cell cycle analysis showed that CMS preconditioning restored S-phase entry (Figure 1E, F), thereby reversing H₂O₂-induced proliferation arrest. Together, these results indicate that CMS preconditioning preserves ADSCs stemness, enhances paracrine function, and dually inhibits apoptotic/necrotic pathways, thereby bolstering cellular stress resistance**.**

### CMS enhances oxidative stress resistance in ADSCs by coordinating apoptosis-antioxidant axis

**Figure 2:**
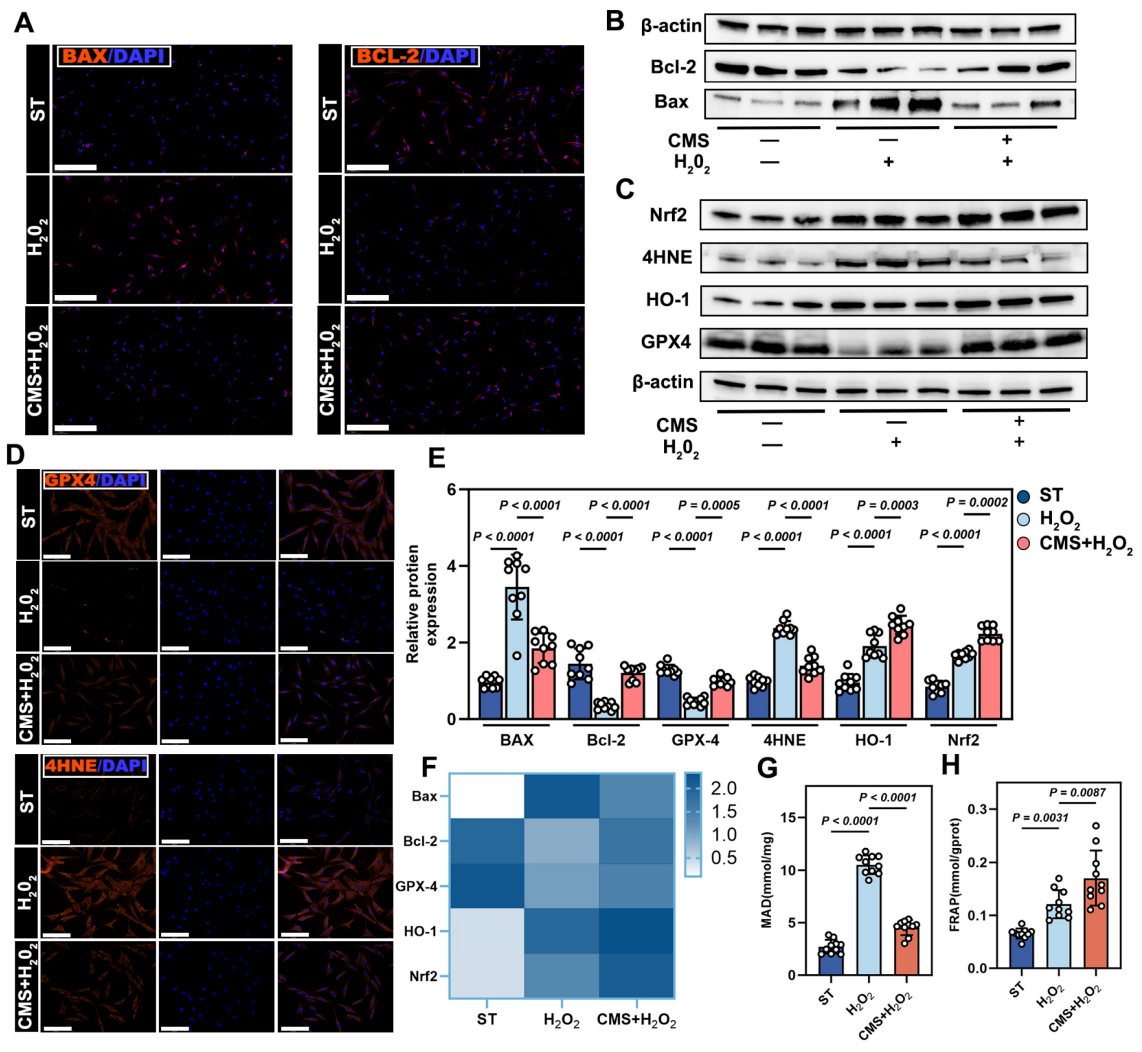
** CMS attenuates oxidative stress-induced ADSCs apoptosis and activates antioxidant pathways.** (A) Immunofluorescence of BAX (red), and Bcl-2 (red) (Scale bar = 125 μm). (B) Western blot analysis apoptosis-related proteins, normalized to β-actin. (C) Western blot analysis Nrf2, HO-1, 4HNE and GPX4 expression, normalized to β-actin. (D) IF of lipid peroxidation marker 4-HNE (red) and glutathione peroxidase GPX4 (red) (Scale bar = 125 μm). (E) Gray values (ImageJ) of BAX, Bcl-2, Nrf2, HO-1, 4HNE and GPX4 (n = 9). (F) BAX, Bcl-2, Nrf2, HO-1, 4HNE and GPX4 mRNA expressions in different ADSCs cells detected by quantitative real-time PCR (n = 9). (G-H) Malondialdehyde and ferric reducing antioxidant power levels in different groups (n =10). (Data are presented as mean ± standard deviation).

Building on the findings that CMS preconditioning dually inhibits apoptotic/necrotic pathways, we next investigated the complementary mechanism by which CMS enhances oxidative stress resistance in ADSCs: its coordination of the apoptosis-antioxidant axis. To elucidate the molecular mechanisms by which CMS mediated oxidative stress resistance, we systematically analyzed the regulation of apoptosis, suppression of lipid peroxidation, and activation of antioxidant defenses. Immunofluorescence and western blot analysis revealed that CMS downregulated pro-apoptotic proteins, BAX, while upregulating anti-apoptotic protein, Bcl-2 (Figure 2A, B). This reversal of pro-/anti-apoptotic protein balance effectively blocked oxidative stress-induced apoptotic cascades, providing critical survival support. Furthermore, CMS markedly reduced the levels of the lipid peroxidation marker, 4HNE, indicating direct protection against membrane oxidative damage.

Mechanistically, given the critical role in Nrf2 signaling antioxidant defense, we assessed its activation 29. CMS robustly activated the antioxidant transcription factor, Nrf2, driving the upregulation of its downstream targets, HO-1 and GPX4 (Figure 2C-F). Biochemical assays confirmed decreased malondialdehyde levels and enhanced total antioxidant capacity (Figure 2G, H). In summary, CMS bolsters ADSCs oxidative stress tolerance through a dual mechanism: (1) suppression of pro-apoptotic signaling to preserve cells survival, and (2) activation of the mechanoresponsive Nrf2-mediated antioxidant system to mitigate oxidative damage. This coordinated regulation of the apoptosis-antioxidant axis underpins ADSCs functional maintenance in pathological microenvironments.

### CMS enhances ROS scavenging capacity and preserves mitochondrial integrity in ADSCs under oxidative stress

**Figure 3:**
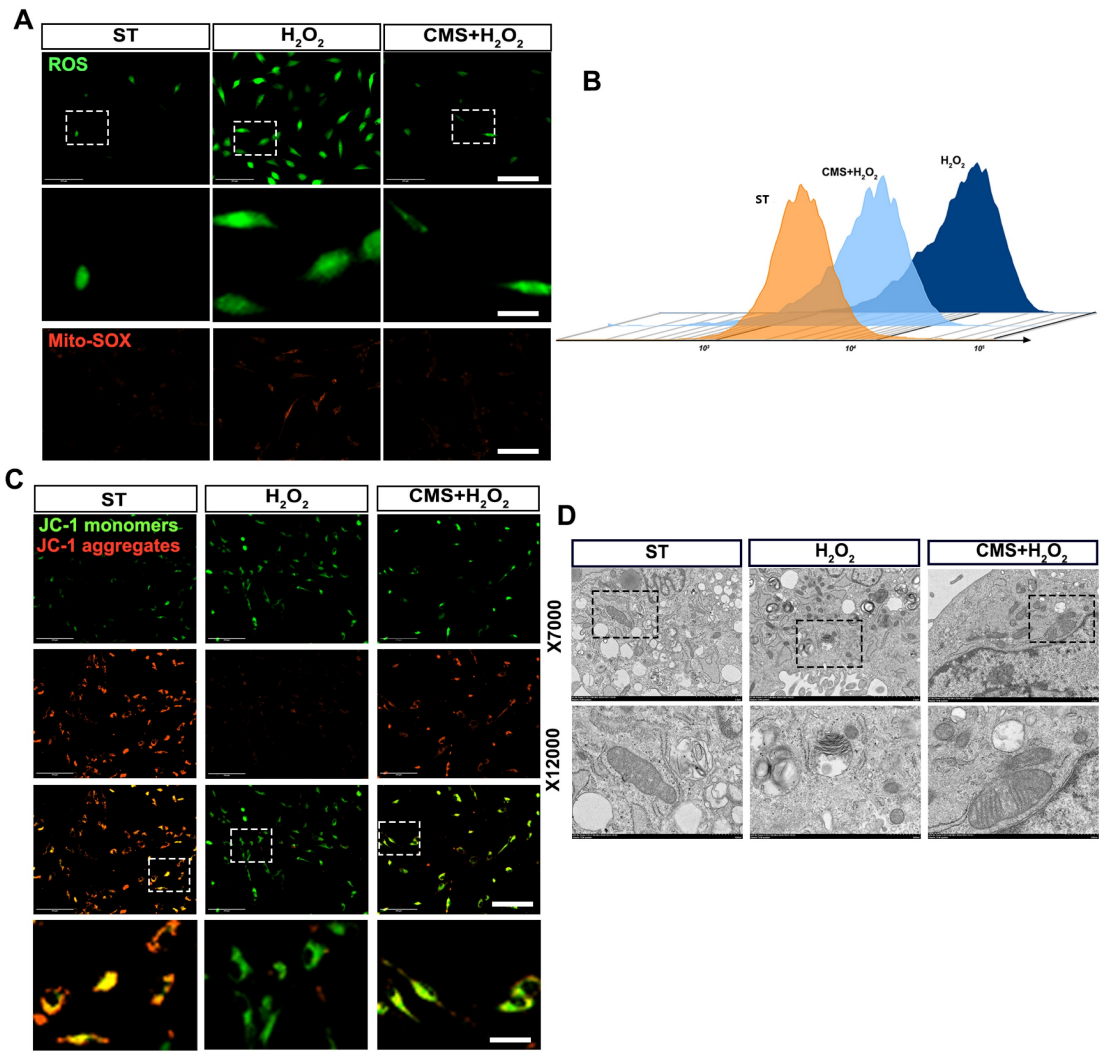
** CMS enhances ROS scavenging capacity and preserves mitochondrial integrity in ADSCs under oxidative stress.** (A-B) DCFH-DA and MitoSOX Red staining were used to assess intracellular and mitochondrial ROS levels, respectively, in ADSCs (Scale bar = 125 μm, Zoom scale bar = 30 μm). (C) JC-1 staining (red: J-aggregates; green: monomers) indicating mitochondrial membrane potential (Scale bar = 125 μm, Zoom scale bar = 30 μm). (D) Transmission electron microscopy images of mitochondrial ultrastructure (Data are presented as mean ± standard deviation).

Based on the observed suppression of apoptosis and enhancement of antioxidant capacity and recognizing that mitochondria are both primary targets of oxidative damage and critical regulators of cellular redox balance and survival, we next investigated the impact of CMS on mitochondrial ROS scavenging and function under oxidative stress. Flow cytometry and immunofluorescence analysis demonstrated reduced total and mitochondrial ROS levels in CMS- preconditioned ADSCs. DCFH-DA and Mito-SOX results revealed that ADSCs exposed to oxidative stress exhibited ROS accumulation, whereas the CMS group exhibited effectively modulated ROS levels (Figure 3A, B). Crucially, since mitochondrial integrity directly dictates cellular bioenergetics and fate under stress, we assessed key functional parameters of mitochondria. JC-1 analysis revealed that CMS preserved high mitochondrial membrane potential (ΔΨm), evidenced by predominant red fluorescence, whereas stressed cells showed depolarization (green fluorescence) (Figure 3C). To determine if the functional protection correlated with structural preservation, we performed TEM analysis. This revealed stark contrasts: mitochondria in the H_2_O_2_ group exhibited cristae fragmentation, reduced matrix density, and swelling, while CMS-treated mitochondria maintained intact cristae with increased density and organized lamellar structures (Figure 3D). These findings demonstrate that CMS preserves mitochondrial ultrastructure and function, thereby enhancing ROS scavenging capacity and maintaining bioenergetic homeostasis—essential for ADSCs survival and function in oxidative environments. The observed structural stabilization suggests CMS may directly influence mitochondrial dynamics through mechanosensitive pathways.

### CMS induces metabolic reprogramming and mitochondrial remodeling in ADSCs

**Figure 4:**
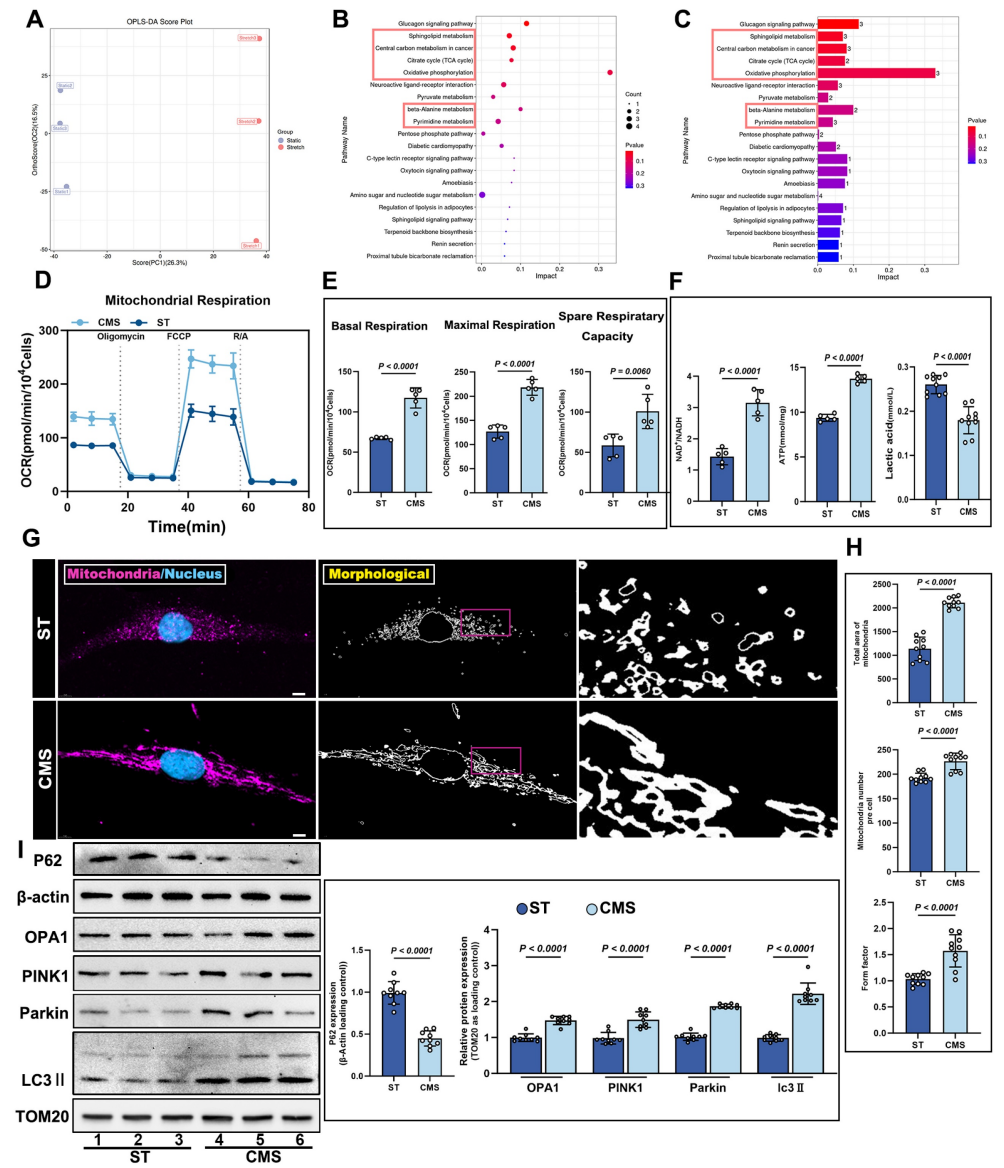
** Non-targeted metabolomics profiling and mitochondrial functional alterations in ADSCs following CMS.** (A) OPLS-DA score plot of Static and CMS-preconditioned ADSCs (n=3 per group). (B-C) KEGG (Kyoto Encyclopedia of Genes and Genomes) pathway enrichment analysis of metabolites with significant changes. (D-E) Representative traces of OCR assay comparing ADSCs under CMS or ST (n = 5). (F) NAD⁺/NADH ratio, ATP, Lactate content in ST and CMS-ADSCs (n = 5). (G) Cultured ADSCs were labeled with MitoTracker Red and Hoechst 33342, and mitochondrial morphological changes were observed using confocal microscopy (Scale bar = 5 μm). (H) Quantitative analysis of the following mitochondrial parameters using ImageJ: mitochondrial number, shape factor [calculated as (Perimeter²)/(4π×Area)], and total mitochondrial area (n = 10). (I) Western blot analysis of OPA1, PINK1, Parkin, LC3II and P62 protein levels in ADSCs under different treatments, with corresponding loading controls (n = 9). (Data are presented as mean ± standard deviation).

Having found that CMS preserves mitochondrial structural integrity and functional capacity under oxidative stress, we next sought to elucidate the systemic regulatory effects of CMS on the metabolic-mitochondrial axis in ADSCs. Specifically, we investigated how CMS leverages this mitochondrial protection to enhance cellular energy homeostasis through coordinated metabolic reprogramming and mitochondrial quality control. Untargeted metabolomic analysis demonstrated that CMS induced substantial metabolic reprogramming in ADSCs. Orthogonal partial least squares-discriminant analysis (OPLS-DA) revealed distinct clustering between statically and mechanically stimulated ADSCs, indicating divergent metabolic profiles (Figure 4A). Kyoto Encyclopedia of Genes and Genomes (KEGG) pathway enrichment analysis revealed that these changes were predominantly associated with the TCA cycle (Figure 4B-C). Seahorse analysis demonstrated that CMS significantly increased the OCR of ADSCs (Figure 4D), accompanied by enhanced basal respiration, maximal respiration, and spare respiratory capacity in ADSCs (Figure 4E). Mechanistically, this bioenergetic adaptation was driven by coordinated activation of key metabolic gatekeepers: Citrate synthase (CS) and pyruvate dehydrogenase (PDH) exhibited substantially augmented activities, directly accelerating mitochondrial substrate flux (Figure S2A, B). Consistent with this enzymatic potentiation, NAD⁺/NADH ratios increased significantly (Figure 4F), reflecting amplified Oxidative phosphorylation (OXPHOS) capacity. Mitochondrial functional analysis further corroborated these effects through elevated ATP production (Figure 4F), where PDH-mediated redirection of pyruvate utilization and CS-catalyzed TCA cycle entry synergistically sustained energy homeostasis. As the primary energy currency, this increase in ATP levels not only reflects enhanced mitochondrial function but also indicates heightened bioenergetic activity in mechanically stimulated cells. Such energy accumulation is critical for cell survival and functional maintenance under oxidative stress. Concurrently, lactate levels decreased (Figure 4F), signifying a metabolic shift from glycolysis dependence to oxidative phosphorylation dominance. Thus, CMS enhances mitochondrial function and bolsters cellular bioenergetic capacity, providing robust support for cell survival and functionality under oxidative stress. Confocal microscopy coupled with morphometric analysis revealed dynamic mitochondrial remodeling in the CMS group, characterized by increased mitochondrial quantity, reduced fragmentation, and enhanced network complexity (Figure 4G, H). This shift may reflect a mitochondrial quality control mechanism-fission-selective autophagy-re-fusion-that eliminates damaged mitochondria while optimizing functional subpopulations. Molecular evidence supports this hypothesis. Western blot analysis demonstrated that CMS significantly upregulated the expression of the mitochondrial fusion protein, OPA1, mitophagy regulators, PINK1/Parkin, autophagy marker LC3II and decreased the expression of autophagy substrate p62 (Figure 4I). Furthermore, TEM revealed that CMS significantly enhanced mitophagy in ADSCs (Figure S2C). These findings indicate that CMS protects mitochondrial function and enhances mitochondrial autophagy while boosting cellular energy production and metabolic adaptability.

### ATP mediates the antioxidant and pro-survival effects of CMS-preconditioned ADSCs

**Figure 5:**
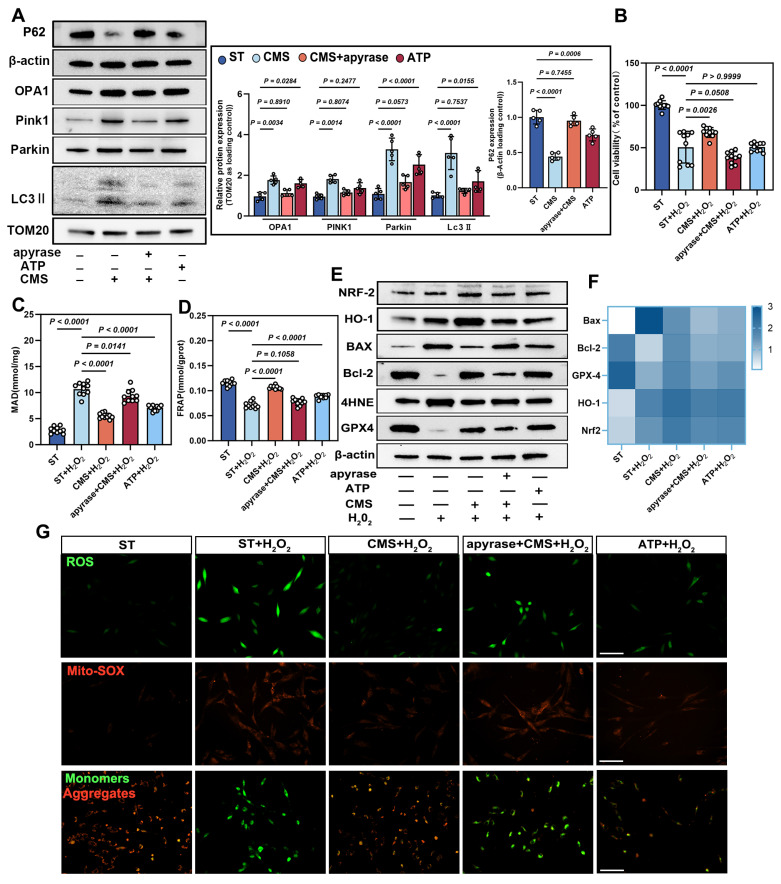
** CMS enhances ADSCs mitophagy and survival under oxidative stress via ATP production.** (A-B) Western blot analysis of OPA1, PINK1, Parkin, LC3II and P62 in the ST, CMS, ATP-depleted (CMS + apyrase), and ATP-supplemented ADSCs. Quantification of protein levels normalized to β-actin and TOM20 (n = 5). (B) Cell Counting Kit-8 assay of control, CMS, CMS + apyrase, and ATP-supplemented ADSCs exposed to H₂O₂ (200 μM, 24 h) (n =10). (C-D) Malondialdehyde and ferric reducing antioxidant power levels in different groups (n = 10). (E) Western blot of BAX, Bcl-2, 4HNE, GPX4, HO-1 and Nrf-2. Quantification of protein ratios normalized to β-actin. (F) qPCR quantification of BAX, Bcl-2, GPX4, HO-1 and Nrf-2. (G) ROS levels (DCFH-DA/Mito-SOX) and mitochondrial membrane potential (JC-1, red: J-aggregates/green: monomers) were assessed by fluorescence imaging, with red/green ratio quantifying ΔΨm (Scale bar = 275 μm). (Data are presented as mean ± standard deviation).

Based on our findings that CMS preserves mitochondrial function, enhances mitophagy, and promotes cellular energy production through metabolic adaptation, we investigated the functional role of ATP, the primary energy output of mitochondrial metabolism, in coordinating CMS-induced cellular protection. We demonstrate that CMS-induced ATP accumulation directly fuels mitochondrial fission and selective autophagic clearance of damaged organelles, while simultaneously sustaining energy homeostasis. This ATP-driven coordination of mitochondrial quality control and bioenergetic stability establishes a mechanoadaptive defense against oxidative stress-induced dysfunction**.** Western blot analysis showed that CMS upregulated OPA1, PINK1, Parkin, and LC3II., and downregulated the protein expression of P62 (Figure 5A), which are key regulators of mitochondrial function and cell survival. Exogenous ATP supplementation replicated these effects, whereas ATP depletion produced the opposite outcomes.

CCK-8 assays further demonstrated that CMS markedly enhanced cell viability under oxidative stress compared to untreated ADSCs (Figure 5B). ATP depletion significantly reduced cell viability, whereas ATP supplementation partially restored survival rates, underscoring the indispensable role of ATP in CMS-mediated cytoprotection. CMS enhanced cellular antioxidant capacity in an ATP-dependent augmentation of redox homeostasis, as evidenced by suppressed MDA levels (Figure 5C) and elevated FRAP (Figure 5D). These effects were mimicked by ATP supplementation and abrogated by ATP depletion these benefits, confirming that bioenergetic modulation governs the redox equilibrium. Western blot and PCR analysis corroborated this finding by showing that ATP supplementation upregulated the expression of anti-apoptotic Bcl-2 and GPX4 while downregulating the expression of BAX and lipid peroxidation marker, 4HNE. Similarly, the expression of HO-1 and NRF-2 was upregulated. Conversely, ATP depletion reversed these trends (Figure 5E, F), further supporting ATP's central role in orchestrating an antioxidant defense network.

Immunofluorescence assays confirmed that CMS significantly attenuated intracellular ROS accumulation under oxidative stress, indicating ATP-dependent ROS and Mito-SOX scavenging (Figure 5G). JC-1 staining revealed that CMS preserved ΔΨm. ATP depletion drastically reduced ΔΨm, while ATP supplementation partially restored it (Figure 5G), suggesting that ATP sustains electron transport chain integrity to minimize electron leakage, thereby enhancing cell survival under oxidative stress.

### Piezo1 serves as the core receptor in the mechano-ATP-mitophagy signaling cascade

**Figure 6:**
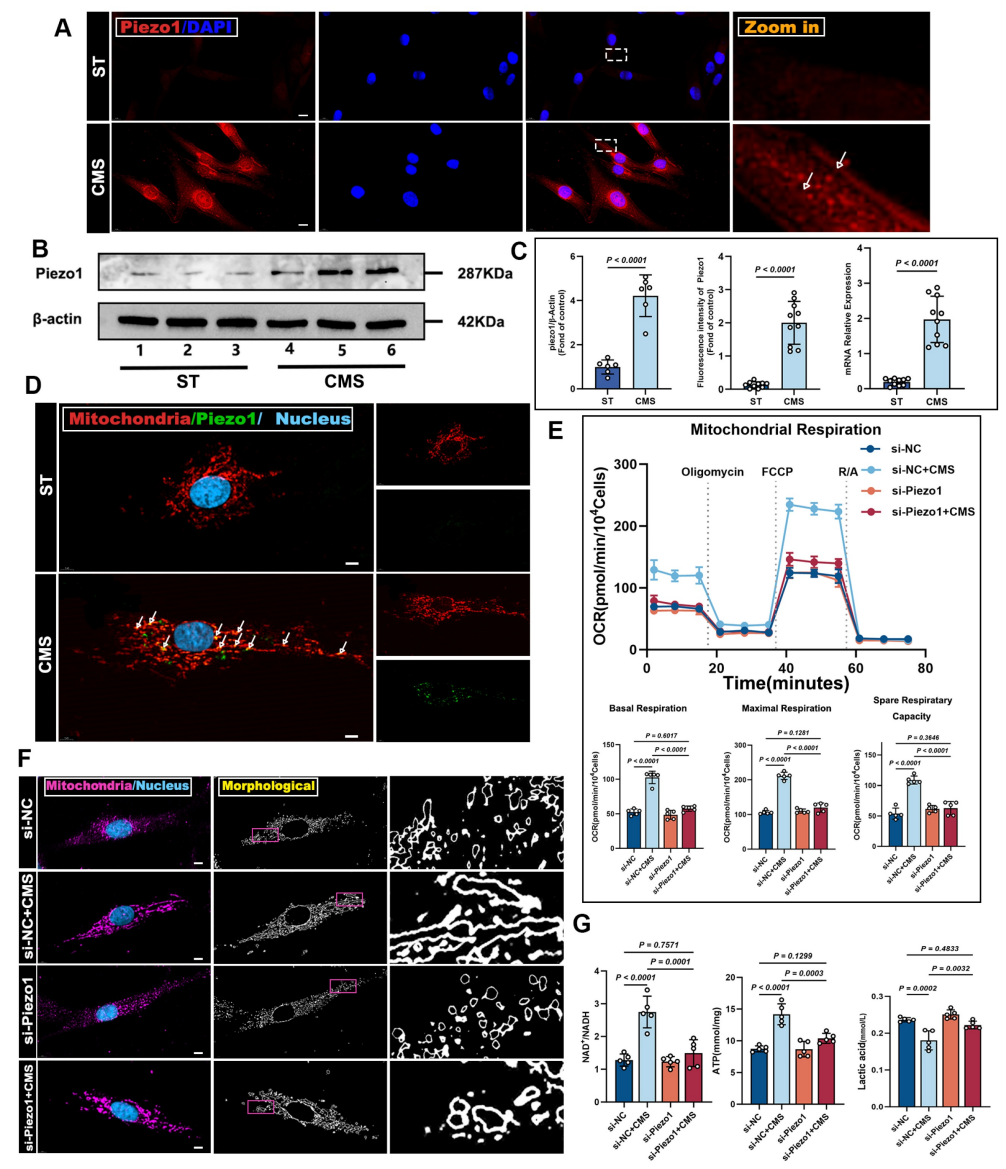
** CMS enhances ADSCs mitochondrial dynamics and mitophagy via Piezo1 upregulation.** (A) Immunofluorescence staining of Piezo1 (red) in ST and CMS ADSCs. Nuclei are counterstained with DAPI (blue) (Scale bar = 10 μm). (B) Western blot showing Piezo1 levels in the ST and CMS groups. (C) Quantitative analysis of Piezo1 using Immunofluorescence (n = 10), Western blot (n = 6), and quantitative real-time PCR (n = 10) (D) Representative Immunofluorescence images of co-localized Piezo1 (green) and mitochondria (red), in CMS-treated ADSCs. Merged images show overlapping signals (yellow) (Scale bar = 5 μm). (E) Representative traces of OCR assay ADSCs in the context of Piezo1 knockdown (n = 5). (F) Cultured ADSCs were labeled with MitoTracker Red and Hoechst 33342, and mitochondrial morphological changes were observed using confocal microscopy in the context of Piezo1 knockdown (Scale bar = 5 μm). (G) NAD⁺/NADH ratio, ATP, Lactate content in ADSCs in the context of Piezo1 knockdown (n = 5). (Data are presented as mean ± standard deviation).

Building upon these findings, we identified Piezo1 as a mechanosensitive channel that governs ADSCs bioenergetic adaptation to mechanical cues. Immunofluorescence staining verified Piezo1 expression during CMS (Figure 6A), hinting at Piezo1-mediated heightened mechanical sensitivity. This was backed by western blot and PCR analysis, which detected much higher Piezo1 protein levels in the CMS group versus the control group (Figure 6B, C). Moreover, we found that Piezo1 was distributed in the plasma membrane and cytoplasm; therefore, we speculated whether it could be expressed in mitochondria. Confocal microscopy demonstrated the co-localization of Piezo1 with the mitochondrial (Figure 6D), indicating that Piezo1 is expressed not only on the plasma membrane but also on mitochondria. These findings imply that Piezo1 may regulate cellular metabolism and survival by modulating mitochondrial function.

**Figure 7:**
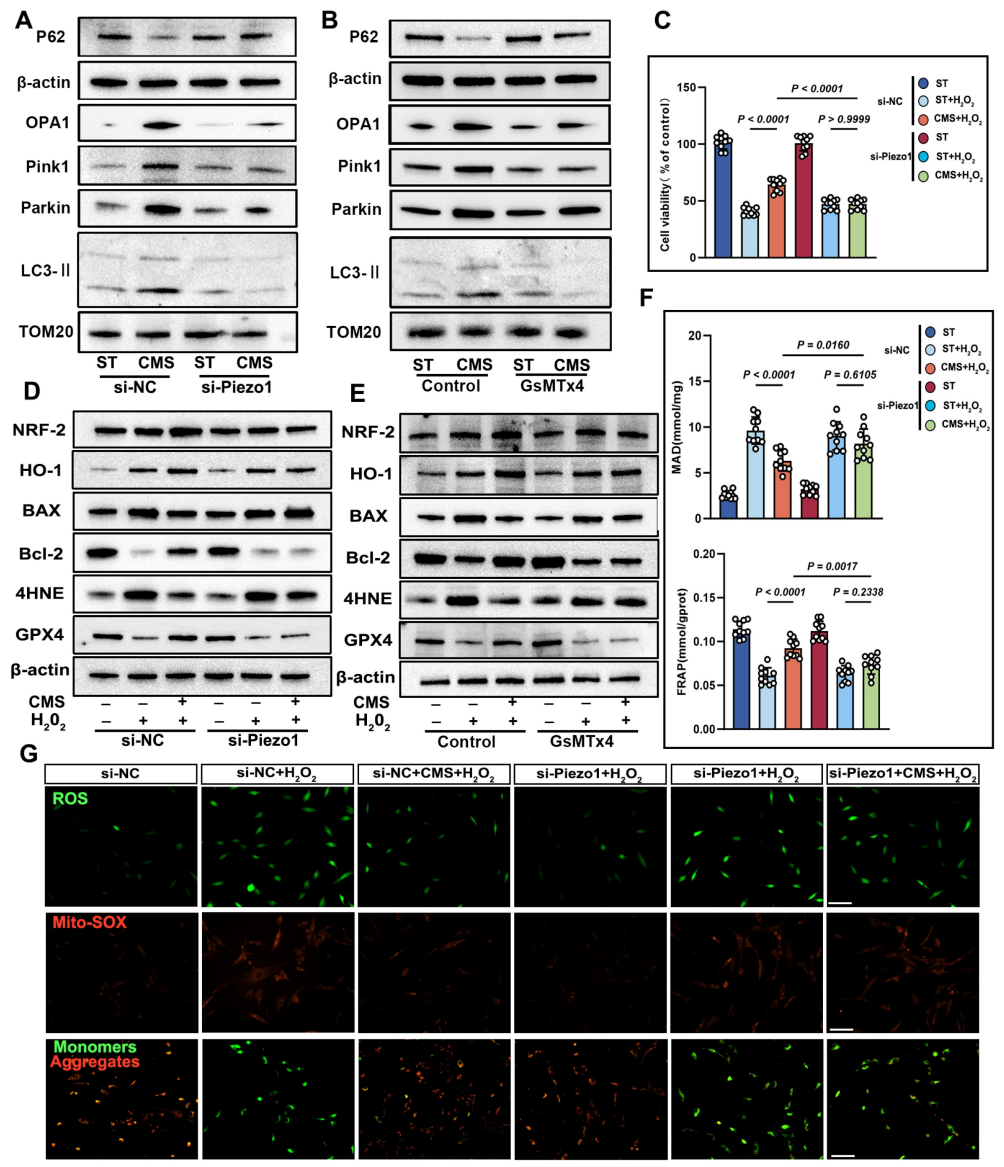
** CMS enhances ADSCs survival and mitochondrial integrity under oxidative stress via Piezo1 upregulation.** (A-B) Western blot analysis of OPA1, PINK1, Parkin, LC3II and P62 was performed under the conditions of Piezo1 knockdown or in the presence of the Piezo1 inhibitor GsMTx4. Quantification of protein levels normalized to β-actin and TOM20. (C) CCK-8 assay was conducted under conditions of Piezo1 knockdown or in the presence of the Piezo1 inhibitor GsMTx4 (n = 10). (D-E) EApoptosis and antioxidant protein expression. Western blot of BAX, Bcl-2, 4HNE, GPX4, HO-1 and Nrf-2. Quantification of protein ratios normalized to β-actin. (F) Malondialdehyde and ferric antioxidant power levels in different groups (n = 10). (G) ROS levels (DCFH-DA/Mito-SOX) and mitochondrial membrane potential (JC-1, red: J-aggregates/green: monomers) were assessed by fluorescence imaging, with the red/green ratio quantifying ΔΨm, under conditions of Piezo1 knockdown (Scale bar = 275 μm). (Data are presented as mean ± standard deviation).

To explore Piezo1's role in mechanical signaling, we knocked down Piezo1 in ADSCs using siRNA. Compared to scrambled siRNA-transfected cells, Piezo1-knocked-down cells showed reduced mitochondrial respiration under CMS (Figure 6E). Crucially, enzymatic activity assays revealed concurrent suppression of core metabolic regulators CS and PDH (Figure S2D, E), which directly contributed to the observed metabolic dysfunction: NAD⁺/NADH and ATP ratios decreased while lactate levels increased due to Piezo1 knockdown (Figure 6G). Similar phenotypes were observed upon treatment with the Piezo1-specific inhibitor GsMTx4 (Figure S3), confirming that Piezo1 is crucial for CMS-induced metabolic reprogramming in ADSCs. Western blot analysis demonstrated that in Piezo1-knockdown cells or cells treated with the Piezo1 inhibitor GsMTx4 under CMS, the expression of OPA1, PINK1, Parkin, and LC3II was inhibited, while the expression of P62 was restored (Figure 7A, B). These results confirm Piezo1's crucial role in mechanically induced mitophagy. Furthermore, oxidative stress resistance conferred by CMS preconditioning-evident in normal ADSCs (CCK8, FRAP, MDA), was abolished when Piezo1 was knocked down (Figure 7C, F). These cells failed to upregulate the anti-apoptotic protein Bcl-2 or the antioxidant enzyme GPX4 in response to CMS preconditioning and could not suppress the pro-apoptotic protein BAX or lipid peroxidation marker 4HNE upon subsequent oxidative challenge (Figure 7D, E). Consistent with this, immunofluorescence and JC-1 staining showed that Piezo1 knockdown impaired the ability of CMS to reduce intracellular/mitochondrial ROS accumulation or restore ΔΨm under oxidative stress (Figure 7G). The Piezo1 inhibitor GsMTx4 similarly blocked the CMS-induced protective phenotype (Figure S4), confirming the essential role of Piezo1 in mediating this adaptive response. In summary, this study identified Piezo1 as the core receptor in the mechano-ATP-mitophagy cascade. By orchestrating ATP production and mitophagy, Piezo1 significantly enhanced ADSCs survival and antioxidant capacity under oxidative stress.

### CMS enhances the *in vivo* survival rate of ADSCs

**Figure 8:**
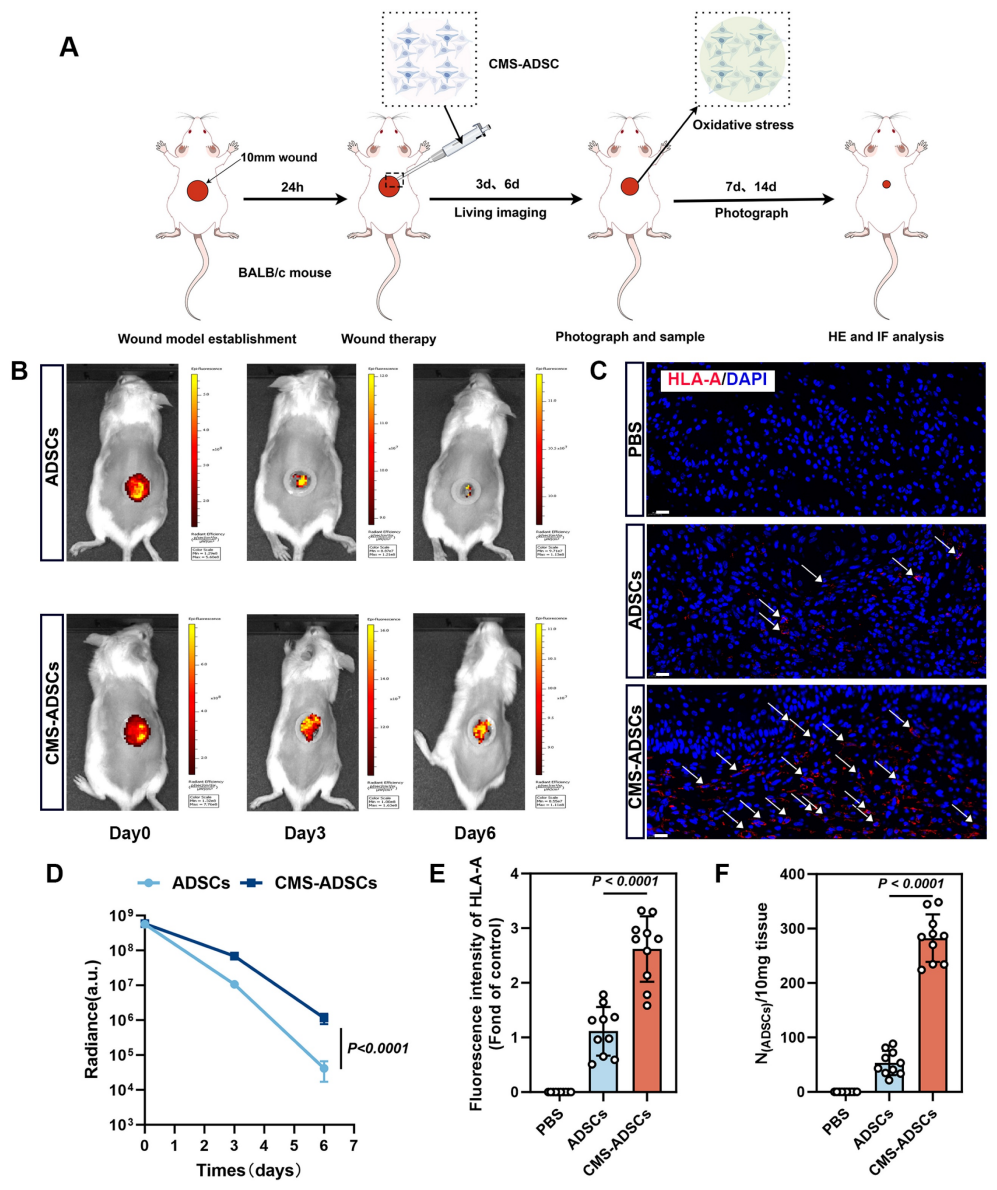
** CMS enhances ADSCss survival in wound healing.** (A) Illustration of wound and the procedural steps on the dorsum of Balb/c mice. (B) *In vivo* bioluminescence monitoring of ADSCs engraftment. Optical signals were quantified in ADSCs-transplanted wounds (5×10⁶ cells/20μ L), and mechanically primed ADSCs-treated wounds (5×10⁶ cells/20 μL) across sequential observation phases. (C, E) Immunofluorescence analysis of HLA-A infiltration in wound tissues at day 6 post-treatment (Scale bar = 20 μm). (D) Real-time *in vivo* luminescence quantification at lesion areas post-treatment across specified time points (n=10). (F) Evaluation of ADSCs retention in the traumatized zones using qPCR analysis 6 days post-administration(n=10). (Data are presented as mean ± standard deviation).

To evaluate the retention and therapeutic efficacy of CMS-ADSCs *in vivo*, we examined the distribution and persistence of transplanted cells. ADSCs were transduced with lentivirus expressing luciferase (Luc) for bioluminescence imaging. A full-thickness excisional wound model (1 cm diameter) was established on the dorsum of the Balb/c mice after hair removal and surgical excision (Figure 8A).

Mice were randomly divided into the following three treatment groups: (1) PBS group (negative control); (2) untreated ADSCs group, and (3) CMS-ADSCs group. Bioluminescence imaging on days 0, 3, and 6 post-transplantations revealed that CMS-ADSCs exhibited significantly stronger signals in the wound bed compared to untreated ADSCs (Figure 8B, D), indicating superior retention and survival. Immunofluorescence staining of human-specific HLA-A in wound tissues revealed significantly higher engraftment efficiency in the CMS-preconditioned ADSC group compared to the PBS control or untreated ADSC group (Figure 8C, E). These findings were further validated by quantitative PCR analysis (Figure 8F), collectively indicating that CMS pretreatment enhanced the survival and retention of ADSCs in the oxidative stress-rich wound microenvironment.

### CMS enhances the ability of ADSCs to accelerate wound healing

**Figure 9:**
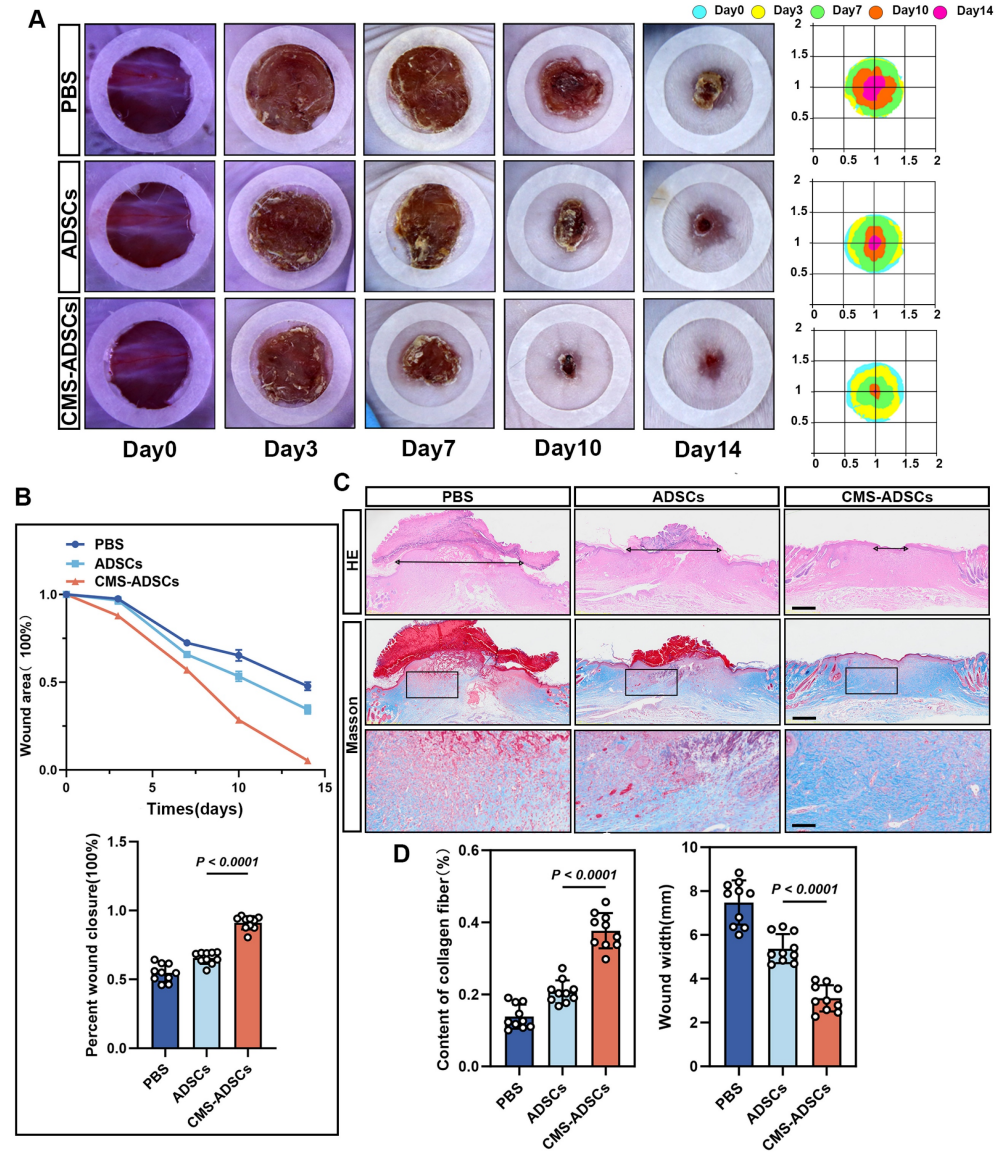
** CMS enhances ADSCs therapeutic efficacy in wound healing.** (A) Representative wound images across the treatment groups. Macroscopic wound appearance at days 0, 3, 7, 10, and 14 post-injuries. (B) Wound closure kinetics and area quantification. Temporal changes in wound area from day 0 to day 14, expressed as percentage of initial wound size. And quantification of wound closure percentage across groups at day 14 (n=10). (C-D) Representative H&E and Masson's trichrome stained sections, along with quantitative analysis of histological features (Scale bars: main panels = 500 μm; Zoom scale bar = 125 μm) (n=10). (Data are presented as mean ± standard deviation).

Therapeutic efficacy was assessed by measuring wound closure rates on days 3, 7, 10, and 14. The CMS-ADSCs group exhibited accelerated wound healing, achieving 50% closure significantly earlier and reaching 95% closure by day 14, whereas the PBS and normal ADSCs groups showed only 52% and 66% closure, respectively (Figure 9A-C). Histological evaluation via H&E and Masson's trichrome stains day 14 revealed complete epidermal stratification, organized collagen fibers, and a mature basement membrane in the CMS-ADSCs group, contrasting with the disorganized ECM and immature structures in the other groups (Figure 9C, D).

### CMS-enhanced ADSCs transplantation improves angiogenesis and alleviates wound inflammation

**Figure 10:**
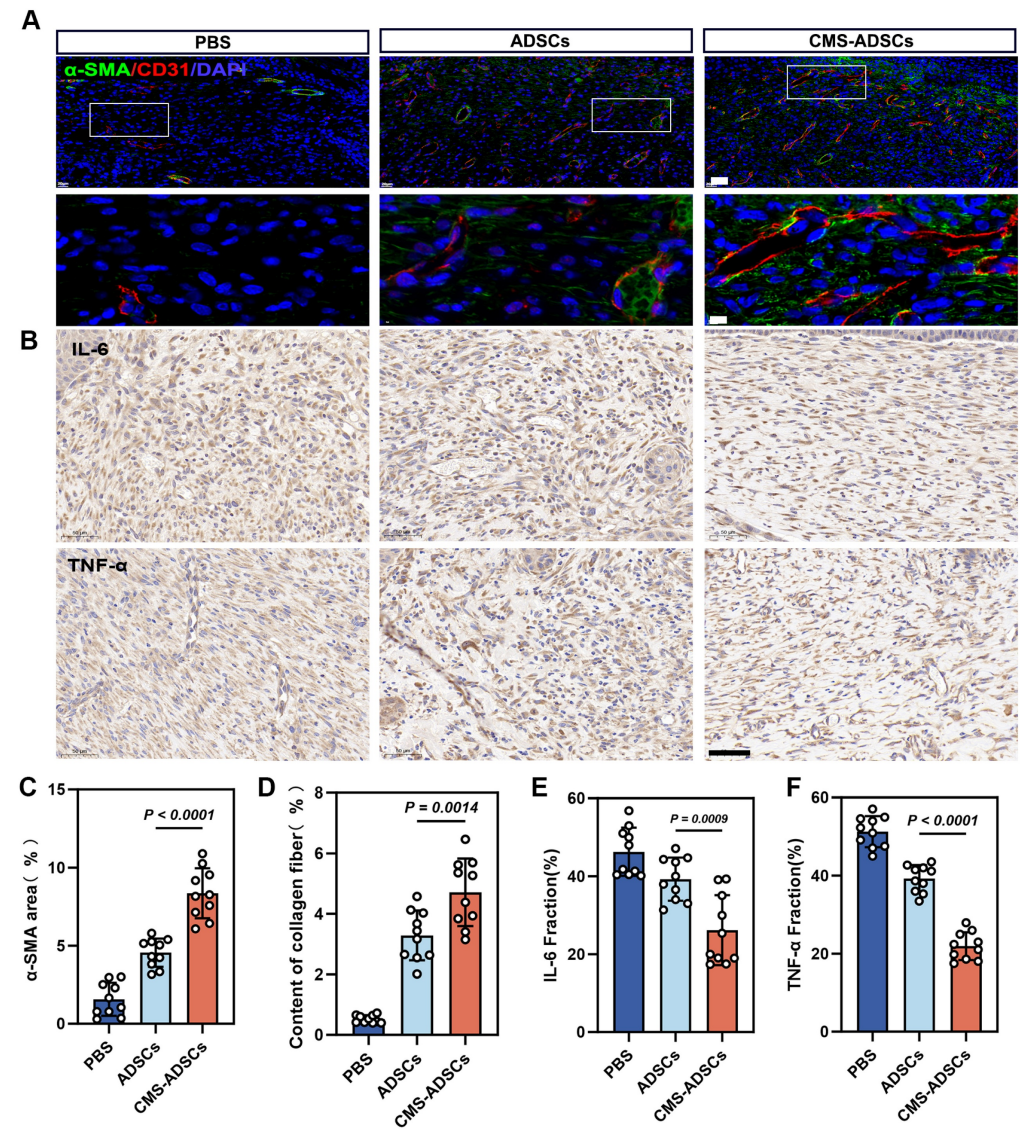
** CMS-ADSCs can simultaneously alleviate the inflammatory response at wound site and promote vascular regeneration.** (A) Fluorescence co-staining images of CD31 (red, endothelial cells) and α-smooth muscle actin (α-SMA, green) in day 14 wound tissues. Nuclei are counterstained with DAPI (blue) (Scale bar = 20 μm, Zoom scale bar = 5 μm). (B) Immunohistochemical engineering staining for IL-6 and TNF-α of skin wound tissue (Scale bar = 50 μm). (C-D) Quantitative analysis of CD31+ area and α-SMA+ vessel density(n=10). (E-F) Quantitative analysis of IL-6 and TNF-α density(n=10). (Data are presented as mean ± standard deviation).

Angiogenesis, a key hallmark of healing, was significantly enhanced in the CMS-ADSC group. Immunofluorescence analysis demonstrated higher expression of CD31 (endothelial marker) and α-SMA (vascular smooth muscle marker), along with mature luminal structures in newly formed vessels (Figure 10A, C, D). Experimental data also revealed that CMS-ADSCs transplantation mitigated the hyperinflammatory response in chronic wounds. IHC analysis showed reduced TNF-α and IL-6 expression in ADSCs-treated wounds compared to controls, demonstrating their ability to promote a phenotypic shift from inflammatory dominance to tissue remodeling (Figure 10B, E, F). These findings indicate that CMS-ADSCs not only prolong the survival time of transplanted cells but also suppress inflammatory responses and synergistically accelerate tissue regeneration. This study provides theoretical and practical support for the optimization of stem cells therapies in clinical settings.

## Discussion

Wound healing is a complex biological process that restores the skin's structural and functional integrity following injury. It progresses through four sequential phases: hemostasis, inflammation, proliferation, and remodeling. During hemostasis, blood coagulation prevents excessive bleeding, whereas the inflammatory phase eliminates pathogens and necrotic debris. The proliferative phase involves tissue regeneration and angiogenesis, followed by remodeling, which restores tensile strength and elasticity via collagen realignment 2. As critical regulators of cellular architecture and functionality, mechanical forces modulate physiological processes across these phases. For instance, during hemostasis and inflammation, mechanical cues facilitate conformational changes in platelets to enhance thrombus formation and accelerate immune cell migration for efficient pathogen clearance 38. Controlled mechanical stimulation promotes keratinocyte and fibroblast proliferation, endothelial differentiation, and granulation tissue formation 39, whereas excessive mechanical stress during remodeling triggers fibroblast hyperactivation, leading to pathological scarring 40. Accordingly, therapeutic strategies such as tissue expansion, tension-reducing devices, and negative-pressure wound therapy, exploit mechanical principles to optimize healing outcomes 41.

In recent years, significant progress has been made in the application of mechanical stimulation in regenerative medicine. Studies have found that different types of mechanical stimuli such as tensile stress, shear stress, compression, and hydrostatic pressure can affect cell proliferation, differentiation, and migration, thereby promoting tissue repair and regeneration 42,43. For example, novel magnetically responsive scaffolds have been developed, which use magnetic nanoparticles as non-contact actuators to activate the hydrogel, causing deformations of different amplitudes and thereby exerting mechanical stimulation on cells. In both *in vitro* and *in vivo* experiments, dynamic mechanical stimulation accelerated the wound healing process by promoting re-epithelial formation and mediating skin contraction 44. Mechanical stimulation can also affect cellular behavior and function by modulating intracellular signaling pathways, such as MAPKs 45.

Among mesenchymal stem cell sources, ADSCs offer unique advantages due to their accessibility, multipotency, and immunomodulatory properties, which represent the cornerstone of regenerative therapies. However, clinical translation remains hindered by rapid functional decline under oxidative stress and instability in mechanically dynamic microenvironments 46. Conventional approaches, such as antioxidant supplementation or genetic engineering, offer transient cytoprotection but are limited by off-target effects and technical complexity 47. Mechanical preconditioning presents a promising, non-invasive biophysical strategy that recalibrates mechanometabolic coupling networks. Recent studies have established that mechanical stimuli modulate ADSCs behavior via mechanosensitive ion channels 17,48.

While He *et al.* demonstrated that CMS-preconditioned ADSCs accelerated wound healing primarily by promoting macrophage polarization toward an anti-inflammatory M2 phenotype through paracrine signaling (notably TSG-6) 17, our study elucidates a fundamentally distinct, cell-intrinsic mechanometabolic cascade centered on mitochondrial resilience and oxidative stress resistance—addressing a critical gap in the field. Although previous studies have primarily focused on cytoskeletal remodeling (e.g., F-actin realignment) or transcriptional regulation (e.g., YAP/TAZ nuclear shuttling), the metabolic and mitochondrial mechanisms underpinning CMS-enhanced ADSCs functionality remain underexplored 49,50. Our findings elucidate a novel mechanometabolic cascade in which CMS activates Piezo1-dependent ATP synthesis, orchestrating multilayered cytoprotection. Specifically, CMS reprograms TCA cycle flux and upregulates OPA1 to amplify PINK1/Parkin-driven mitophagy, thereby enhancing OXPHOS efficiency for sustained ATP production. Under oxidative stress, this metabolic resilience suppresses BAX/BCL2-mediated apoptosis while the central role of Nrf2 in coordinating CMS-induced cytoprotection has also been clearly demonstrated. As a primary modulator of antioxidant defense, CMS activates Nrf2 to directly coordinate the two-arm protection strategy. That is, to control redox homeostasis by upregulating HO-1 and GPX4, while effectively neutralizing ROS and maintaining mitochondrial membrane integrity. Furthermore, our data (e.g., ROS levels, ATP assays, mitophagy markers) indicate possible links between this pathway and the modulation of mitochondrial function, including effects on ATP synthesis and mitophagy processes, which warrant further investigation to clarify their specific contributions to the observed cytoprotection.

This intrinsic enhancement of ADSCs viability and function represents a key mechanistic difference from the paracrine-mediated macrophage modulation described 17. CMS establishes a self-reinforcing mitochondrial quality control cycle. The establishment of this self-reinforcing mitochondrial cycle is a central novelty of our work. This cycle maintains mitochondrial homeostasis in oxidative microenvironments, sustains cellular viability, and enhances therapeutic efficacy. The benefits are achieved through CMS-induced paracrine activation—e.g., VEGF for angiogenesis and TGF-β for immunomodulation—which synergizes with apoptosis suppression to strengthen ADSCs resilience. Crucially, preserved differentiation potential ensures CMS-preconditioned ADSCs retain full regenerative capacity. This integrated mechanism directly enhances ADSCs therapeutic function in hostile microenvironments. We propose a paradigm-shifting "mechano-ATP axis," wherein Piezo1-mediated mechanosensing stimulates ATP synthesis, and ATP reciprocally enhances mitochondrial fusion (via OPA1) and mitophagy, forming a self-sustaining circuit. This bidirectional regulation underpins the enduring effects of transient mechanical preconditioning, in which metabolic memory is encoded by ultrastructural adaptations (e.g., mitochondrial cristae densification) that augment the OXPHOS capacity post-stimulation. Such metabolic imprinting aligns with the emerging evidence linking mechanical cues to epigenetic modifications. Nevertheless, further studies are needed to elucidate these interactions 51.

Mechanical preconditioning of ADSCs offers distinct advantages over pharmacological and genetic strategies. It is non-invasive, scalable, and compatible with smart biomaterials (e.g., stress-responsive hydrogels and piezoelectric scaffolds) for in situ mechanical modulation 52. In our wound healing models, preconditioned ADSCs resist oxidative damage in inflammatory niches while enhancing angiogenesis and collagen remodeling, thereby addressing both structural and functional deficits. For mechanically inaccessible sites (e.g., deep tissue injuries), localized ATP delivery systems mimic mechanometabolic benefits, highlighting the translational versatility of this approach.

Despite these advances, several challenges remain. First, mechanical parameters (e.g., frequency, strain magnitude) require optimization to avoid ferroptosis risk from mechanical overload or insufficient pathway activation due to weak stimulation 53. Second, inherent single-cell metabolic heterogeneity complicates the achievement of consistent therapeutic outcomes. Third, a critical gap persists in bridging *in vitro* mechanical loading models with the dynamic *in vivo* mechanical microenvironment-specifically, tissue stiffness gradients and interstitial shear forces. Future research should prioritize (1) spatiotemporal mapping of metabolic fluxes to decode mechanosignaling dynamics, (2) elucidating mechanoepigenetic crosstalk (e.g., lncRNA-mitochondrial interactions) sustaining metabolic memory, and (3) developing multiscale biomimetic platforms to replicate human mechanopathology.

## Conclusions

This study reveals a mechanometabolic signaling axis by which CMS enhances the oxidative stress resistance of ADSCs, offering a shift from passive cell delivery to active, mechanobiology-guided cellular optimization in regenerative medicine. Specifically, we demonstrated that CMS activates the Piezo1-mediated ATP signaling cascade to coordinate a dual-cell protective program, including: (1) inhibiting mitochondrial apoptosis through BAX/Bcl-2 rebalancing while activating the Nrf2-HO-1/GPX4 antioxidant network to neutralize ROS; and (2) enhancing TCA cycle flux to enhance OXPHOS efficiency, coupled with PINK1/Parkin driven mitochondrial phagocytosis to eliminate damaged mitochondria and further resist oxidative stress. By integrating mechanobiology, metabolomics, and materials science, we propose a novel therapeutic strategy-engineering mechanical microenvironments to precondition cells and customizing their metabolic phenotypes to achieve precise tissue regeneration. This coordinated response establishes a self-reinforcing circuit, maintaining paracrine function through metabolic elasticity, allowing ADSCs to thrive in harsh microenvironments. These findings highlight a promising therapeutic approach for engineering stem cells with improved resilience and regeneration potential via biochemical modulation.

## Supplementary Material

Supplementary figures and table.

## Abbreviations

ADSC: adipose-derived stem cell
MSC: mesenchymal stem cell
CMS: cyclic mechanical stretching
OCR: oxygen consumption rate
ECM: extracellular matrix
EdU: 5-Ethynyl-2'-Deoxyuridine
FGF-2: fibroblast growth-2
FITC: fluorescein isothiocyanate
FRAP: Ferric ion reducing antioxidant power
MDA: malondialdehyde
PBS: phosphate-buffered saline
PFA: paraformaldehyde
AO: acridine orange
PI: propidium iodide
ROS: reactive oxygen species
H&E: hematoxylin and eosin
TEM: transmission electron microscopy
siRNA: small interfering RNA
CS: citrate synthase
PDH: pyruvate dehydrogenase
TCA: tricarboxylic acid
OXPHOS: oxidative phosphorylation
KEGG: Kyoto Encyclopedia of Genes and Genomes
OPLS-DA: orthogonal partial least squares-discriminant analysis
TNF-α: tumor necrosis factor-alpha
VEGF: vascular endothelial growth factor
IHC: immunohistochemistry
