# Supplementary material for: Cyclic mechanical stretching enhances mitophagy and oxidative stress resistance in adipose-derived stem cells via the Piezo1/ATP axis to accelerate wound healing
Source: Theranostics. 2025 Sep 8;15(18):9862–84. doi: 10.7150/thno.118364 (PMC12486429; doi:10.7150/thno.118364)
Supplement: Supplementary file 1 — Supplementary figures and table. [file thnov15p9862s1.pdf]

Table S1: Primer and siRNA sequences

Gene	Sequence (5'-3')	
Bax	F: CCCGAGAGGTCTTTTTCCGAG	R: CCAGCCCATGATGGTTCTGAT
Bcl2	F: GGTGGGGTCATGTGTGTGG	R: CGGTTCAGGTACTCAGTCATCC
GXP4	F: GAGGCAAGACCGAAGTAAACTAC	R: CCGAACTGGTTACACGGGAA
HO-1	F: AAGACTGCGTTCCTGCTCAAC	R: AAAGCCCTACAGCAACTGTCG
Nrf2	F: GGTTGGCCCTTTCCTGCTTTATA	R: CCCTTGACAGCACAGAAGAGAAT
Piezo1	F: ACTCCGCACTCATCAAGTGG	R: CAGCAGGAGAAAGTCGCTGA
HLA-A	F: TTCTGGAGAGGAGCAGAGATACA	R: CTATCTGAGCTCTTCCTCCTCCA
β-actin	F: CATGTACGTTGCTATCCAGGC	R: CTCCTTAATGTCACGCACGAT
Piezo1-siRNA	S: CCAAGUACUGGAUCUAUGU	AS: ACAUAGAUCCAGUACUUGG

F: Forward; R: reverse; S: sense; AS: antisense

Figure S1

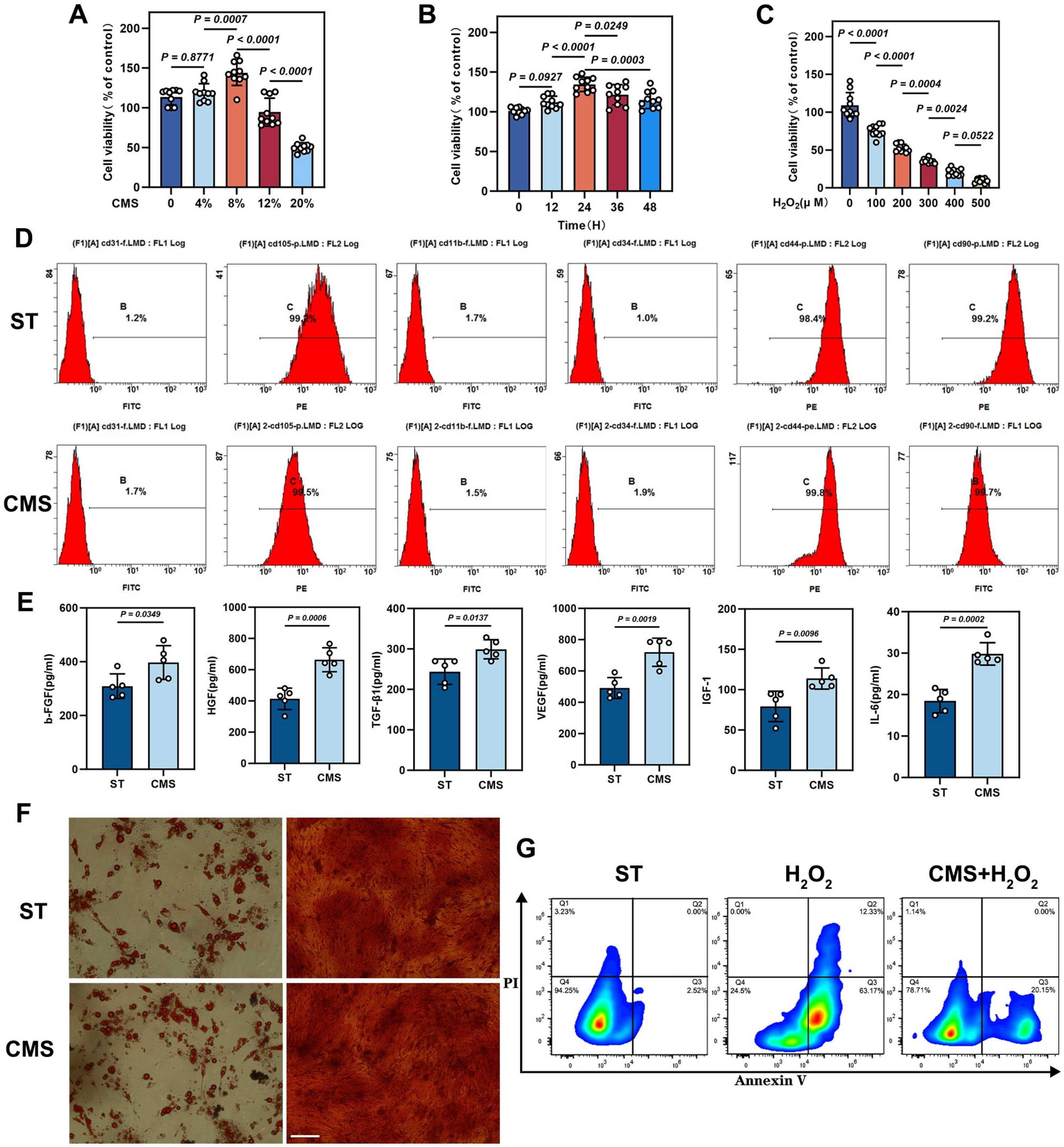
A
Cell viability (% of control)
CMS
0
4%
8%
12%
20%
P = 0.0007
P < 0.0001
P = 0.8771
P < 0.0001
B
Cell viability (% of control)
Time (H)
P = 0.0249
P < 0.0001
P = 0.0003
P = 0.0927
C
Cell viability (% of control)
H_2O_2(μ M)
P < 0.0001
P < 0.0001
P = 0.0004
P = 0.0024
P = 0.0522
D
ST
CMS
FITC
PE
E
b-FGF(pg/ml)
HGF(pg/ml)
TGF-β1(pg/ml)
VEGF(pg/ml)
IGF-1
IL-6(pg/ml)
P = 0.0349
P = 0.0006
P = 0.0137
P = 0.0019
P = 0.0096
P = 0.0002
ST
CMS
F
ST
CMS
G
ST
H_2O_2
CMS+H_2O_2
PI
Annexin V

Fig. S1. Effects of Cyclic Mechanical Stretch (CMS) and oxidative stress on viability, phenotype, paracrine function, and apoptosis of adipose-derived stem cells (ADSCs).

(A) Cell Counting Kit-8 (CCK-8) proliferation assay of ADSCs subjected to varying mechanical strain amplitudes (0%, 4%, 8%, 12%, 20%) (n=10). (B) CCK-8 assay assessing ADSC proliferation after CMS for different durations (12 h, 24 h, 36 h, 48 h) (n=10). (C) H2O2 dose-response curve (0, 100, 200, 300, 400, 500 μM; 24 h) showing ADSC viability assessed via CCK-8 assay (n=10). (D) Flow cytometry identification of surface markers for ADSCs cultured under static conditions (ST) and after CMS. (E) Comparison of paracrine secretion profiles of ADSCs under CMS or ST conditions (n=5). (F) Representative images comparing adipogenic and osteogenic differentiation potential of ADSCs under CMS or ST conditions (Scale bar = 275 μm). (G) Apoptosis analysis of ADSCs assessed by flow cytometry using Annexin V/PI staining. (Data are presented as mean ± standard deviation).

Figure S2

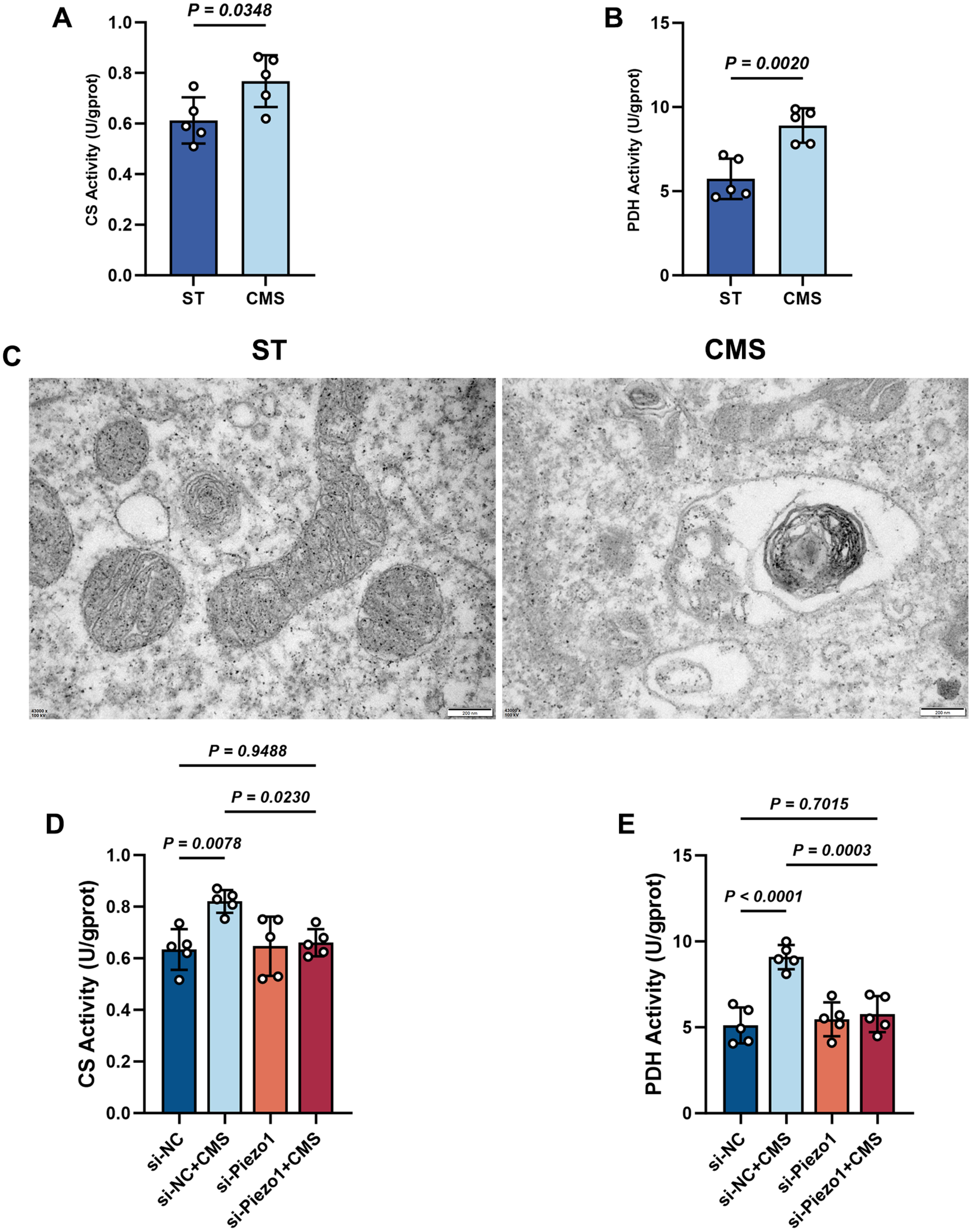

A
P = 0.0348
CS Activity (U/gprot)
1.0
0.8
0.6
0.4
0.2
0.0
ST
CMS
B
PDH Activity (U/gprot)
15
10
5
0
P = 0.0020
ST
CMS
C
ST
CMS
D
P = 0.9488
P = 0.0230
P = 0.0078
CS Activity (U/gprot)
1.0
0.8
0.6
0.4
0.2
0.0
si-NC
si-NC+CMS
si-Piezo1
si-Piezo1+CMS
E
P = 0.7015
P = 0.0003
P < 0.0001
PDH Activity (U/gprot)
15
10
5
0
si-NC
si-NC+CMS
si-Piezo1
si-Piezo1+CMS

Fig. S2. Effects of CMS and Piezo1 Knockdown on Citrate Synthase (CS) and Pyruvate Dehydrogenase (PDH) Levels in ADSCs

(A-B) CS and PDH levels in ADSCs cultured under CMS versus ST (n=5).
(C) Transmission electron microscopy images of mitochondrial ultrastructure (n=5). (D-E) CS and PDH content in ADSCs following Piezo1 knockdown (n=5). (Data are presented as mean ± standard deviation).

Figure S3

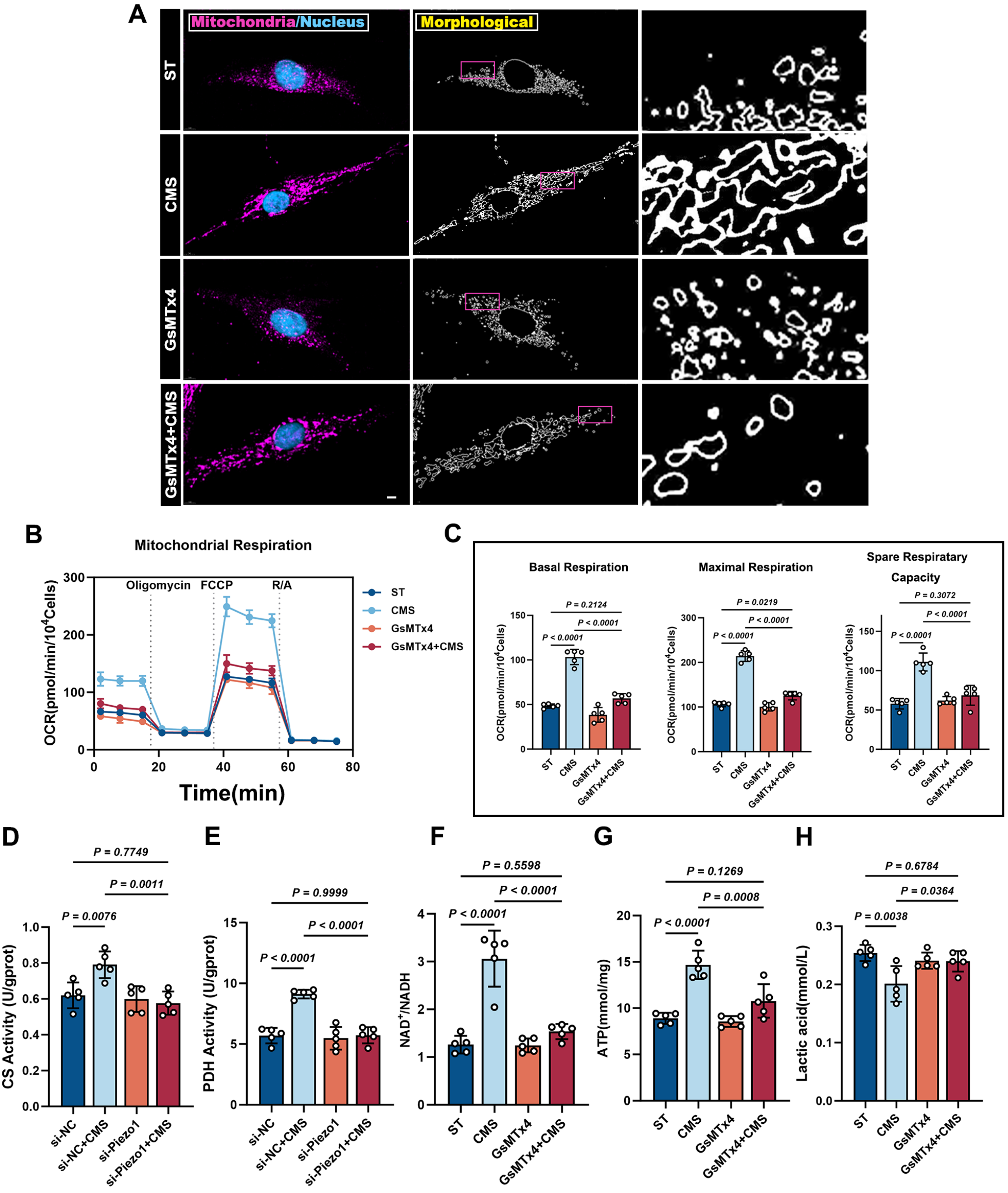
A
Mitochondria/Nucleus
Morphological
ST
CMS
GsMTx4
GsMTx4+CMS
B
Mitochondrial Respiration
Oligomycin
FCCP
R/A
OCR(pmol/min/10⁴Cells)
Time(min)
ST
CMS
GsMTx4
GsMTx4+CMS
C
Basal Respiration
Maximal Respiration
Spare Respiratary Capacity
P = 0.2124
P < 0.0001
P < 0.0001
P = 0.0219
P < 0.0001
P < 0.0001
P = 0.3072
P < 0.0001
P < 0.0001
D
P = 0.7749
P = 0.0011
P = 0.0076
CS Activity (U/gprot)
si-NC
si-NC+CMS
si-Piezo1
si-Piezo1+CMS
E
P = 0.9999
P < 0.0001
P < 0.0001
PDH Activity (U/gprot)
F
P = 0.5598
P < 0.0001
P < 0.0001
NAD⁺/NADH
G
P = 0.1269
P = 0.0008
P < 0.0001
ATP(mmol/mg)
H
P = 0.6784
P = 0.0364
P = 0.0038
Lactic acid(mmol/L)

Fig. S3. Effect of Piezo1 inhibitor GsMTx4 on mechanical stimulation-enhanced ADSCs mitochondrial dynamics and mitophagy.

(A) Cultured ADSCs were labeled with MitoTracker Red and Hoechst 33342, and mitochondrial morphological changes were observed using confocal microscopy in the presence of the Piezo1 inhibitor GsMTx4 (Scale bar = 5 μm). (B-C) Representative traces of OCR assay ADSCs in the presence of the Piezo1 inhibitor GsMTx4 (n=5). (D-H) CS, PDH, ATP, NAD^{+}NADH, MAD and FRAP levels in ADSCs in the presence of the Piezo1 inhibitor GsMTx4 (n=5). (Data are presented as mean ± standard deviation).

Figure S4

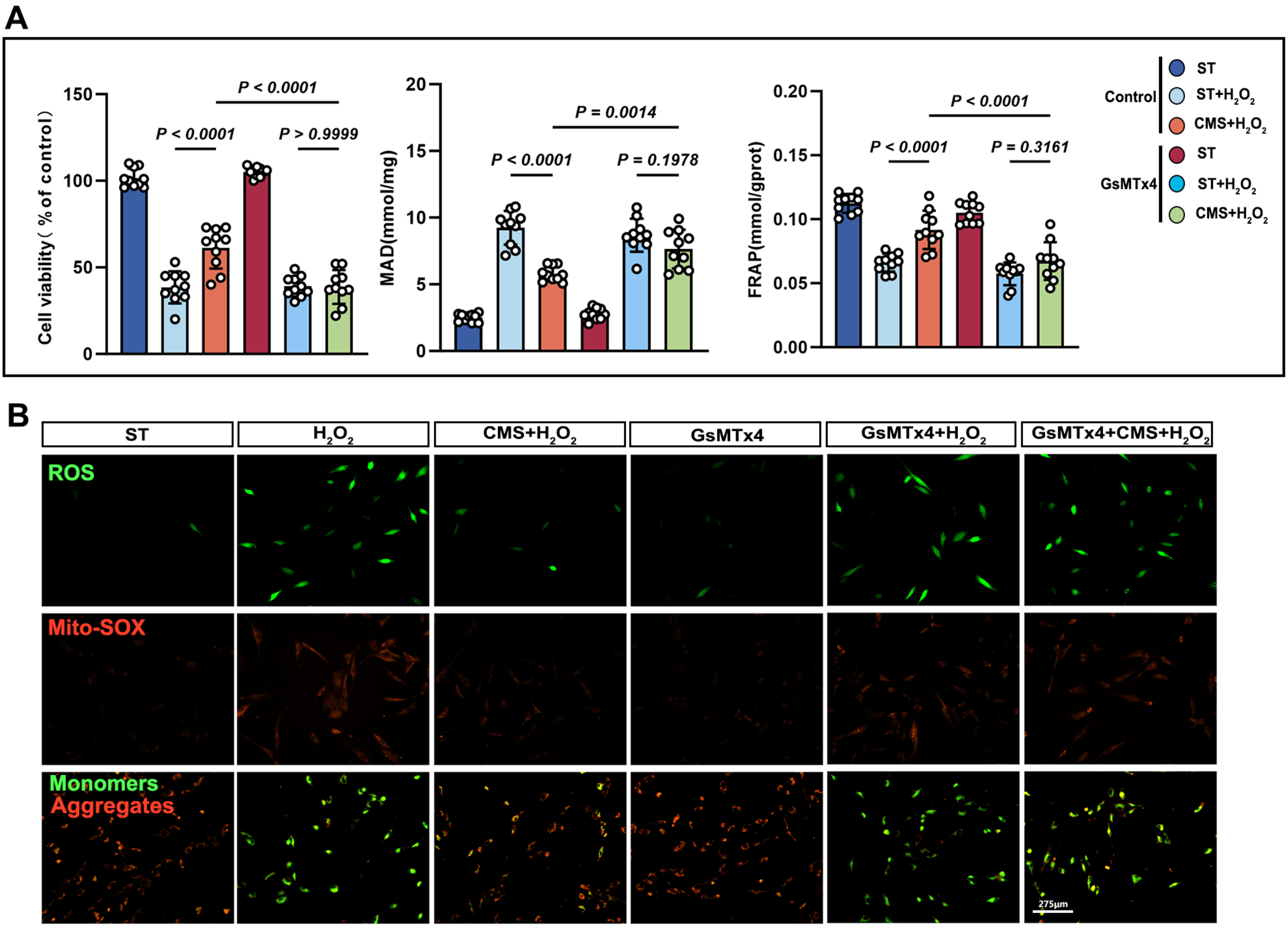

Fig. S4. Effect of Piezo1 inhibitor GsMTx4 on mechanical stimulation-enhanced survival and mitochondrial homeostasis of ADSCs under oxidative stress.

(A) CCK-8 assay and measurements of malondialdehyde and ferric reducing antioxidant power levels were conducted in the presence of the Piezo1 inhibitor GsMTx4 (n=10). (B) ROS levels (DCFH-DA/Mito-SOX) and mitochondrial membrane potential (JC-1, red: J-aggregates/green: monomers) were assessed by fluorescence imaging, with the red/green ratio quantifying ΔΨm, in the presence of the Piezo1 inhibitor GsMTx4(Scale bar = 275 μm). (Data are presented as mean ± standard deviation).
